# Investigation of the Biological Activities and Characterization of Bioactive Constituents of *Ophiorrhiza rugosa* var. *prostrata* (D.Don) & Mondal Leaves through In Vivo, In Vitro, and In Silico Approaches

**DOI:** 10.3390/molecules24071367

**Published:** 2019-04-08

**Authors:** Md. Adnan, Md. Nazim Uddin Chy, A.T.M. Mostafa Kamal, Md Obyedul Kalam Azad, Arkajyoti Paul, Shaikh Bokhtear Uddin, James W. Barlow, Mohammad Omar Faruque, Cheol Ho Park, Dong Ha Cho

**Affiliations:** 1 Department of Bio-Health Technology, Kangwon National University, Chuncheon 24341, Korea; mdadnan1991.pharma@gmail.com (M.A.); azadokalam@gmail.com (M.O.K.A.); chpark@kangwon.ac.kr (C.H.P.); 2 Senior Scientist, Rentia Plant Factory, Chuncheon 24341, Korea; 3 Department of Pharmacy, International Islamic University Chittagong, Chittagong 4318, Bangladesh; nazim107282@gmail.com; 4 Drug Discovery, GUSTO A Research Group, Chittagong 4000, Bangladesh; arka.bgctub@gmail.com; 5 Head of Research and Technology, Rentia Plant Factory, Chuncheon 24341, Korea; 6 Department of Microbiology, Jagannath University, Dhaka 1100, Bangladesh; 7 Ethnobotany and Pharmacognosy Lab, Department of Botany, University of Chittagong, Chittagong 4331, Bangladesh; bokhtear@cu.ac.bd (S.B.U.); omf@cu.ac.bd (M.O.F.); 8 Department of Chemistry, Royal College of Surgeons in Ireland, D02YN77 Dublin, Ireland; jambarlow@rcsi.ie

**Keywords:** *Ophiorrhiza rugosa*, Rubiaceae, antidiarrheal, anti-inflammatory, anthelmintic, antibacterial, in silico molecular docking, ADME/T and PASS

## Abstract

*Ophiorrhiza rugosa* var. *prostrata* is one of the most frequently used ethnomedicinal plants by the indigenous communities of Bangladesh. This study was designed to investigate the antidiarrheal, anti-inflammatory, anthelmintic and antibacterial activities of the ethanol extract of *O. rugosa* leaves (EEOR). The leaves were extracted with ethanol and subjected to in vivo antidiarrheal screening using the castor oil-induced diarrhea, enteropooling, and gastrointestinal transit models. Anti-inflammatory efficacy was evaluated using the histamine-induced paw edema test. In parallel, in vitro anthelmintic and antibacterial activities were evaluated using the aquatic worm and disc diffusion assays respectively. In all three diarrheal models, EEOR (100, 200 and 400 mg/kg) showed obvious inhibition of diarrheal stool frequency, reduction of the volume and weight of the intestinal contents, and significant inhibition of intestinal motility. Also, EEOR manifested dose-dependent anti-inflammatory activity. Anthelmintic action was deemed significant (*P* < 0.001) with respect to the onset of paralysis and helminth death. EEOR also resulted in strong zones of inhibition when tested against both Gram-positive and Gram-negative bacteria. GC-MS analysis identified 30 compounds within EEOR, and of these, 13 compounds documented as bioactive showed good binding affinities to M3 muscarinic acetylcholine, 5-HT3, tubulin and GlcN-6-P synthase protein targets in molecular docking experiments. Additionally, ADME/T and PASS analyses revealed their drug-likeness, likely safety upon consumption and possible pharmacological activities. In conclusion, our findings scientifically support the ethnomedicinal use and value of this plant, which may provide a potential source for future development of medicines.

## 1. Introduction

Due to ongoing reports of antibiotic resistance by various pathogens, researchers have refocused their interest in the use of natural antimicrobial agents to treat infections instead of established antibiotics [1]. Although some conventional antibiotics may be bactericidal, they remain unable to inhibit the release of bacterial toxins which complicates the clinical picture [2]. Analogous to bacterial infections, helminths can persistently infect both humans and animals throughout their lifespan. Helminths exhibit greater complexity than other pathogens and are capable of producing chronic disease, yet these diseases are often neglected in developing regions [3]. The association of bacteria and parasites with gastrointestinal disorders is a common situation in developed and developing countries. Various etiological (*Salmonella*, *Campylobacter*, *Escherichia*, *Shigella*, *Yersinia enterocolitica*, parasites, and viruses) agents responsible for enteric infections may lead to dysentery-like chronic diarrhea [4]. Such infectious diseases cannot be cured easily at present, due to rapid resistance to available drugs; therefore screening of new therapeutic avenues such as plants may provide an alternate and effective approach for the development of novel agents.

Throughout the ages, plants have served humans for innumerable therapeutic interventions, ranging from the common cold to life-threatening conditions. The value of phytomedicinal approaches still resonates with the R&D departments of modern pharmaceutical giants [5]. The development of modern medicines has, in many instances, stemmed from ethnic medicinal uses, and meticulous investigation of naturally occurring bioactive compounds derived from plant screening programs assists the development of new synthetic drugs [6]. As plant-derived drugs contain a pool of metabolites with potential complementary pharmacological actions, their use in mitigating chronic diseases through synergism is an area of intense interest [7]. In this light, indigenous knowledge can help to contribute to the rational drug discovery and development of new drugs from medicinal plants [8]. While indigenous communities typically have a rich knowledge of ethnic medicines, these uses are based on empirical evidence, and proper mechanistic knowledge of biological or pharmacological properties necessitates a scientifically sound investigation, followed by the documentation and characterization of bioactive components of the studied species [9,10]. Hence, proper research on medicinal plants is invaluable in the search for novel bioactive agents for the management of the disease. Cognizant of these principles, we selected the ethnomedicinal plant *Ophiorrhiza rugosa* var. *prostrata* for the present study. 

*Ophiorrhiza rugosa* var. *prostrata* (D.Don) Deb & Mondal (syn: *Ophiorrhiza harrisiana B.Heyne ex Hook.f, Ophiorrhiza prostrata D.Don*) is an annual herb belonging to the Rubiaceae family, which naturally grows in Chittagong and both the Chittagong Hill Tract and Sylhet regions of Bangladesh, where it is variously known as ‘Jari’ or ‘kalashona’ (Chakma), ‘Jariphul’ (Tanchangya) or ‘Pahari mehedi’ (Marma). *O. rugosa* var. *prostrata* is used by the Tanchangya, Marma, and Chakma indigenous communities for the treatment of different diseases. For example, a paste of the leaves is used for the treatment of skin infections (boils) by the Tanchangya people. The Marma community prepares a tea from the leaves, which is drunk daily for the treatment of body aches and chest pain, while the Chakma community applies sun-dried crushed leaves to the ears for the treatment of earache (personal communication). In addition, the crushed roots of the plant are used for the treatment of dysentery [11,12]. Juice from the leaves is drunk in the treatment of diarrhea within the Marma community (personal communication). However, despite such widespread use, there has been no scientific investigation to date on either pharmacological or phytochemical aspects of the plant to validate its traditional uses. Therefore, we aimed to investigate the bioactive components of *O. rugosa* var. *prostrata* leaves using gas chromatography-mass spectrometry (GC-MS). As plants contain a mixture of phytochemicals, robust separation and identification methods are important to elucidate potential bioactive and toxic constituents [13]. GC-MS, coupled with appropriate detection systems is an invaluable tool for the separation and identification of the components of complex, volatile mixtures [14]. Many plant secondary metabolites are sufficiently small, adequately volatile, and thermostable in the GC environment to be easily analyzed by GC-MS [15]. In addition to the phytochemical investigation, we aimed to investigate the known therapeutic applications of the plant through a combination of in vivo (antidiarrheal and anti-inflammatory), in vitro (anthelmintic and antibacterial) and in silico (molecular docking, ADME/T and PASS) analyses.

## 2. Results

### 2.1. GC-MS Analysis 

**Figure 1:**
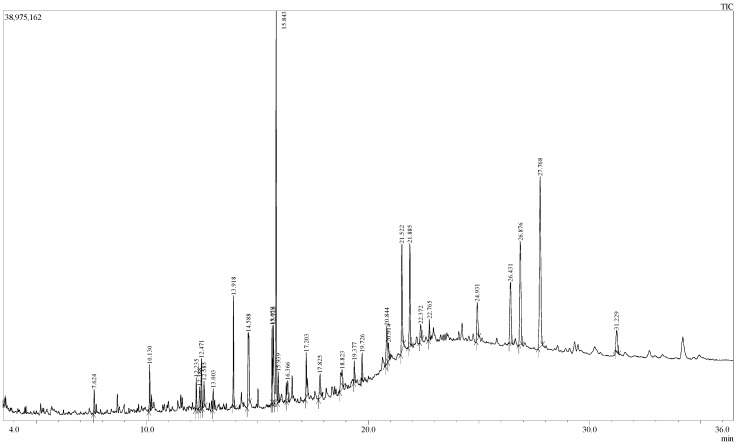
Total ionic chromatogram (TIC) of EEOR (GC-MS, 70eV).

**Figure 2:**
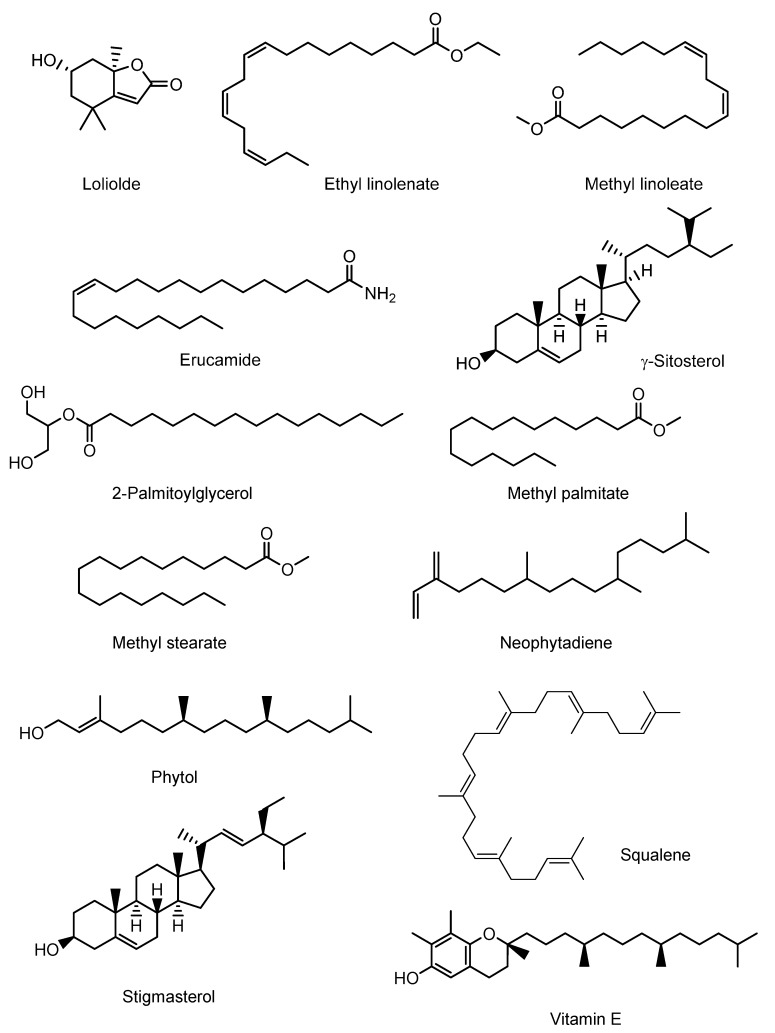
Chemical structures of the major bioactive compounds identified in the EEOR.

The GC-MS analysis of EEOR revealed 30 compounds, which are listed in Table 1, along with their chemical composition, while the total ionic chromatogram (TIC) is shown in Figure 1. The most abundant component by peak area was shown to be phytol (15.50%), followed by γ-sitosterol (14.94%), stigmasterol (7.92%), erucamide (5.39%), squalene (4.83%), methyl palmitate (3.95%), methyl linoleate (2.96%), vitamin E (2.51%), methyl stearate (1.21%), ethyl linolenate (1.17%), loliolide (1.10%), 2-palmitoylglycerol (0.91%), and neophytadiene (0.86%). The structures of these compounds are presented in Figure 2.

### 2.2. Acute Toxicity Test

The acute oral toxicity testing of EEOR did not show any particular evidence of toxicity or behavioral abnormalities at doses of 5, 50, 100, 200, 400, 1000 or 2000 mg/kg. During the 72 h inspection period, no mortality or physical changes such as allergic reactions, loss of body weight, etc. were observed at the specified doses (data not shown).

### 2.3. Qualitative Phytochemical Screening

Preliminary phytochemical screening of EEOR suggested the presence of alkaloids, carbohydrates, flavonoids, phenols, tannins, saponins, steroids, sterols, quinones, oxalate, coumarins, and terpenoids (data not shown).

### 2.4. Effects of EEOR on Castor Oil-Induced Diarrhea in Mice

The effects of EEOR administration on castor oil-induced diarrhea are summarized in Table 2. In this model, EEOR caused significant inhibition of diarrhea, in a dose-dependent manner. The maximum inhibitory effect was observed at a dose of 400 mg/kg (62.50%, *P* < 0.001), which is similar to the reference drug loperamide (65.62%, *P* < 0.001). In addition, EEOR caused a noticeable reduction in defecation numbers at doses of 100 mg/kg (45.20%, *P* < 0.01), 200 mg/kg (52.05%, *P* < 0.001) and 400 mg/kg (60.27%, *P* < 0.001) respectively, compared to the negative control. The reduction of diarrheal feces was also exhibited dose-dependently, with the best antidiarrheal effect observed at the higher dose of 400 mg/kg, compared to the standard drug.

#### 2.4.1. Effects of EEOR on Castor Oil-Induced Enteropooling in Mice

The effect of EEOR on castor oil-induced enteropooling (Table 3) was a significant reduction in the volume and weight of the intestinal contents. In comparison to the negative control (0.51 ± 0.025), the mean volume of intestinal fluids decreased dose-dependently (0.440 ± 0.014, 0.40 ± 0.017 and 0.34 ± 0.063 at doses of 100, 200 and 400 mg/kg EEOR respectively). In addition, compared to the standard drug loperamide, the dose of 400 mg/kg showed a maximal inhibitory effect on both volume (32.29%, *P* < 0.05) and weight (49.57%, *P* < 0.001) of intestinal contents.

#### 2.4.2. Effects of EEOR on Charcoal-Induced Intestinal Transit in Mice

The outcomes following different doses of EEOR on intestinal transit are shown in Table 4. In contrast to the negative control, EEOR significantly (*P* < 0.001) reduced the peristalsis index at three different doses. Of these, the 400 mg/kg dose produced the most significant inhibition (58.33%) of intestinal motility, comparable to the standard drug loperamide (57.73%).

### 2.5. Effects of EEOR on Histamine-Induced Mouse Paw Edema

The anti-inflammatory activity of EEOR and diclofenac sodium against histamine-induced edema is shown in Table 5. The results show that the standard drug significantly (*P* < 0.001) inhibited the inflammatory response (42.42%, 60.29%, 66.66% and 78.57%, respectively, at 1 h intervals for 4 h) after sub-plantar injection of histamine, compared to the control group. On the other hand, oral administration of EEOR (100–400 mg/kg) significantly blocked the inflammatory response induced by histamine in a dose-dependent manner, with a dose of 400 mg/kg displaying statistically significant 38.38%, 42.64%, 54.76% and 57.14% (*P* < 0.001) reductions in paw edema at all hourly intervals over 4 h.

### 2.6. Anthelmintic Activity

**Figure 3:**
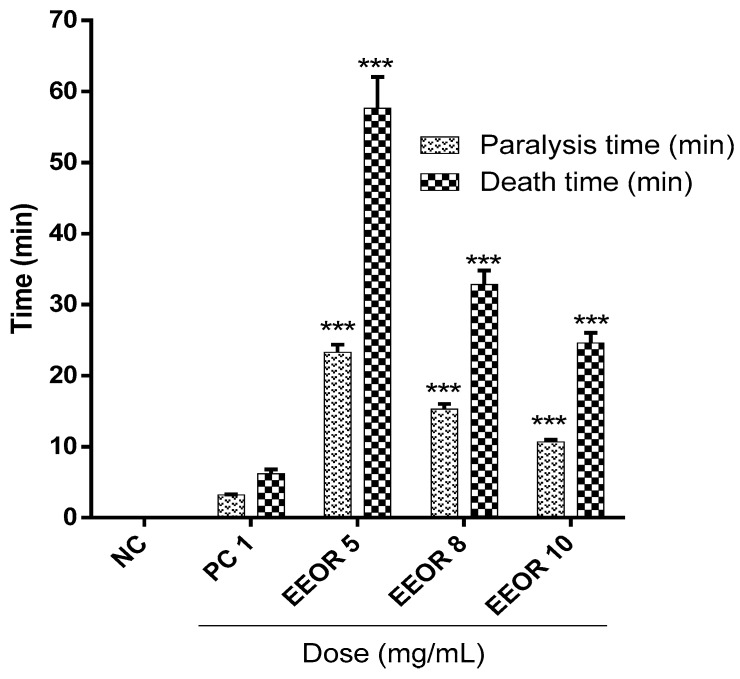
Anthelmintic activity of the ethanol extract of *Ophiorrhiza rugosa* leaves (EEOR). Each value in the table is represented as mean ± SEM (*n* = 3); NC: Negative control; PC: Positive control, Levamisole (1 mg/mL). *** *P* < 0.001 compared with PC (Dunnett’s test).

Figure 3 represents the anthelmintic activity of EEOR. The degree of anthelmintic activity shown by the extract was found to be directly proportional to the concentration employed, ranging from the lowest to highest concentration (5, 8, and 10 mg/mL). At concentrations of 5, 8 and 10 mg/mL, EEOR showed significant (*P* < 0.001) paralysis times of (23.28 ± 1.07), (15.30 ± 0.72) and (10.67 ± 0.31) min, while times to death were (57.63 ± 4.42), (32.83 ± 1.95) and (24.59 ± 1.43) min respectively. In the experiment, the positive control (levamisole, 1 mg/mL) showed a paralysis time of (3.22 ± 0.08) min and time to death of (6.19 ± 0.61) min.

### 2.7. Antibacterial Activity

The antibacterial activity of EEOR is presented in Table 6. The most potent inhibitory effects were exhibited against one Gram-positive (*Bacillus subtilis*), and two Gram-negative (*Salmonella typhi and Escherichia coli*) bacteria. The broadest zone of inhibition (16.23 ± 0.68 mm) was found against *Escherichia coli* at a concentration of 1000 μg/disc, followed by *Bacillus subtilis* (14.80 ± 0.72 mm) and *Salmonella typhi* (12.80 ± 0.34 mm). On the other hand, the extract showed no inhibitory effect against four bacteria, namely *Staphylococcus aureus*, *Bacillus cereus*, *Salmonella paratyphi*, and *Pseudomonas aeruginosa*.

### 2.8. Molecular Docking Study for Antidiarrheal Activity

Results of docking analyses for antidiarrheal activity are shown in Table 7, and the docking figures are shown in Figures S1–S5. In this study, two major receptors (M3 muscarinic acetylcholine receptor, PDB: 4U14; and 5-HT3 receptor, PDB: 5AIN) involved in intestinal motility were used to explore the possible antidiarrheal activity of EEOR. In the case of the M3 muscarinic acetylcholine receptor (PDB: 4U14), Vitamin E showed the highest docking score (−8.80 kcal/mol), better than the standard drug loperamide (−7.32 kcal/mol). On the other hand, for the 5-HT3 receptor (PDB: 5AIN), loliolide (−5.47 kcal/mol) exhibited the highest docking score, followed by ethyl linolenate, phytol, methyl linoleate, neophytadiene, methyl palmitate, and methyl stearate.

Analysis of the docking fits of each compound suggested various interactions between the ligands and the target enzymes. Loliolide interacts with the M3 muscarinic receptor through one H-bond to Asn507 and two π-π stacking interactions with Tyr529 and Tyr533 (docking score −6.63 Kcal/mol). Ethyl linolenate interacts with the same enzyme through the formation of two H-bonds with Ile222 and Leu225 residues (docking score −6.76 kcal/mol), while methyl linoleate interacted with the enzymatic pocket by establishing one H-bond with Ile222 (docking score −3.26 kcal/mol). 2-Palmitoylglycerol interacted through two H-bonds with Asn152 and Ser151 (docking score −3.55 kcal/mol). Methyl palmitate (score: −2.00 kcal/mol), phytol (score: −3.62 kcal/mol), and vitamin E (score: −8.80 kcal/mol) each form one H-bond, with Tyr148, Ile222 and Ser151 residues respectively.

On the other hand, loliolide binds to the enzymatic pocket of the 5-HT3 receptor (PDB ID: 5AIN) by forming one hydrogen bond with Ile116 (docking score −5.47 kcal/mol). Ethyl linolenate (score: −3.47 kcal/mol) and methyl linoleate (score: −1.65 kcal/mol) interact with the same enzymatic pocket, via one H-bond with Glu191 and Arg57 respectively. Methyl stearate interacts with this same enzymatic pocket, by forming one H-bond with Arg57, with a docking score +1.62 kcal/mol. Phytol interacts with the same enzymatic pocket by stabilizing one H-bond with Thr34 (docking score −2.08 kcal/mol). However, methyl palmitate and neophytadiene did not show any interactions with 5AIN. The standard drug loperamide interacts with 4U14 by forming two π-π stacking interactions with Trp525, with a docking score of −7.32 kcal/mol Figure S10C,F.

### 2.9. Molecular Docking Study for Anthelmintic Activity

Results of docking analysis for anthelmintic activity are presented in Table 7. From the results, it is clear that stigmasterol showed the highest docking score against tubulin (−7.13 kcal/mol), followed by γ-sitosterol (−7.00 kcal/mol), vitamin E (−6.65 kcal/mol), ethyl linolenate (−5.36 kcal/mol), loliolide (−4.49 kcal/mol), erucamide (−2.35 kcal/mol), phytol (−2.30 kcal/mol), methyl linoleate (−1.87 kcal/mol), methyl palmitate (−1.10 kcal/mol), and neophytadiene (−0.59 kcal/mol). Among all compounds, three, namely stigmasterol (−7.13 kcal/mol), γ-sitosterol (−7.00 kcal/mol), and vitamin E (−6.65 kcal/mol) showed better docking scores in comparison to the standard levamisole (−6.26 kcal/mol). However, 2-palmitoylglycerol and squalene did not dock with tubulin (PDB: 1XFF). In this study, the best fits found for illustrating the interactions with tubulin are shown in Figures S6 and S7. Ethyl linolenate and methyl linoleate interact with tubulin by forming one H-bond with Lys254, whereas erucamide interacted with the same pocket by establishing one H-bond with Asn101. Methyl palmitate and phytol instead form one H-bond with Lys254. Five compounds, namely loliolide, γ-sitosterol, neophytadiene, stigmasterol, and vitamin E did not show any interactions with tubulin. The docking figures of standard drugs are shown in Figure S10B,E.

### 2.10. Molecular Docking Study for Antibacterial Activity

Thirteen compounds of EEOR were docked with the GlcN-6-P synthase enzyme to assess possible antibacterial activity. Our results indicated that loliolide had the highest binding affinity with the GlcN-6-P synthase enzyme, with a docking score of −4.88 kcal/mol, followed by ethyl linolenate (−3.10), erucamide (−1.21), 2-palmitoylglycerol (−1.16), phytol (−0.12), methyl linoleate (0.25), neophytadiene (+1.18), methyl palmitate (+1.81) and methyl stearate (+2.76). Among all compounds, loliolide and ethyl linolenate showed the best binding affinity against 1XFF, with docking scores of −4.88 and −3.10 kcal/mol respectively, which also ranked better than the standard drug kanamycin (−2.73 kcal/mol). In the antibacterial docking study, the best fit found for loliolide in the enzymatic pocket of GlcN-6-P synthase involved stabilization through the formation of four H-bonds with Hie86, Cyt1, Trp74 and Gly99. The best-ranked fit of ethyl linolenate to the same enzyme was to the binding pocket of 1XFF through two H-bonds to Thr76, and one H-bond with each of Arg73, Asp123, and His77. Methyl linoleate interacts with 1XFF by forming three H-bonds with Arg73, Hie86, and Thr76. The best fit for erucamide in the same enzymatic pocket involved stabilization through the formation of four H-bonds, with Hie86, Arg73, His77, and Asp123. 2-Palmitoylglycerol interacts with the enzyme by forming two H-bonds with Thr76, and two H-bonds with Asp123 and His77. Methyl palmitate interacts with 1XFF via only one H-bond with Arg73, and methyl stearate by forming two H-bonds, with Hie86 and Arg73. Finally, phytol binds to the enzymatic pocket of 1XFF by forming two H-bonds with Asp123 and Thr76. On the other hand, neophytadiene did not show any interactions with 1XFF. The reference drug kanamycin interacts with GlcN-6-P synthase by forming three H-bonds with Trp74, Cyt1, and Gly99, with a docking score of −2.73 kcal/mol. The docking scores obtained for each compound are shown in Table 7, and the docking figures are shown in Figures S8 and S9. The docking figures of standard drugs are shown in Figure S10A,D.

### 2.11. ADME Analysis

According to Lipinski’s rule of five, the compounds γ-sitosterol, squalene, stigmasterol, and vitamin E violated rules of lipophilicity and molecular refractivity. Conversely, loliolide and 2-palmitoylglycerol met Lipinski’s conditions, which are considered to predict optimal drug-like character. All other compounds contravened no more than one rule (Table 8).

### 2.12. PASS Prediction

PASS analysis indicated possible targets and likely pharmacological activities of each of the major compounds within EEOR. We evaluated six biological properties for each compound, based on the values of Pa > Pi and Pa > 7. This prediction approach suggested several important activities of the compounds studied, including antibacterial, anthelmintic, anti-inflammatory, spasmolytic and antiprotozoal actions, which are relevant to our present study. The predicted pharmacological activity profiles of all major compounds are presented in Table 9.

## 3. Discussion

Infectious and parasitic diseases continue to represent intimidating issues for developing countries, due to the lack of useful and safe drugs and the increasing resistance of pathogens to available antibiotics or anti-parasitic agents. A common manifestation of these issues is infectious diarrhea, attributable to both enteric bacterial pathogens and parasites [16]. Such infectious agents may evoke not only adverse effects on intestinal functions but also increase systemic risk via compromising host immunity, leading to increased morbidity and mortality [17]. To treat such infectious diseases, different plant parts, plant extracts, and plant-derived products have been used in traditional medicine. However, many of these traditional medicines have not been formally reported in the literature to date. Recent comprehensive reports on plants used for the treatment of infectious diseases, including diarrhea and dysentery have indicated their possible applications as alternative therapies [18]. In Ethiopia for example, a range of medicinal plants including *Calpurnia aurea*, *Croton marcostachyus*, and *Echinops kebercho* have been scientifically validated as anti-infective agents [19]. In addition, combined screening of anti-diarrheal and anti-infective properties of medicinal plants could prove a valid strategy to identify novel therapeutics. A study conducted by *Taylor* et al. 2013 suggested that plants demonstrating significant anti-bacterial activity against entero-pathogens could be considered as potential diarrheal treatments [20]. In vitro and in vivo investigation of *Rhus* plants including *Rhus semialata*, *Rhus javanica*, and *Rhus tripartitum* produced significant anti-bacterial and antidiarrheal effects and the authors concluded that the presence of antibacterial agents might mediate the diarrhea prevention [21,22]. However, to recognize the intrinsic value of plant extracts, the involvement of both in vitro and in vivo approaches is important in the clinically search for effective anti-infective agents. Studies of plants with established ethnomedicinal uses must consider ethnomedicinal preparation practices when evaluating materials scientifically in the laboratory environment. Thorough extraction protocols are important to completely evaluate both therapeutic and toxicological potential of medicinal plants. Typically, plant phytochemicals possess diverse chemical functionalities, yet most are readily soluble in methanol or ethanol, due to their high extractability and high polarity. Many nonpolar compounds are also soluble in this solvent [23,24]. Therefore methanol and ethanol are frequently used for extraction of medicinal plants prior to evaluation of their therapeutic potential, and we selected ethanol for our extraction of *O. rugosa* leaves, the most commonly used part of the plant. Our study identified potential novel active components from the ethanol extract of *Ophiorrhiza rugosa* leaves (EEOR), having antidiarrheal, anti-inflammatory, anthelmintic and antibacterial properties.

To verify the ethnomedicinal uses of *Ophiorrhiza rugosa*, we examined its antidiarrheal activity, as well as its possible mechanism(s) of action in different animal diarrheal models. In all diarrheal experiments, a high dose of the natural laxative castor oil (0.5 mL) was administered to each mouse. The active metabolite of the oil (ricinoleic acid) is liberated via the action of small intestinal lipases, thus altering the motility of gastrointestinal smooth muscle [25,26]. Upon binding of the metabolite with EP3 prostanoid receptors on smooth muscle cells, it inhibits water and electrolyte absorption from the intestine, resulting in accumulation of fluid and interruption of secretory functions, which in turn generates a deleterious effect in the intestine [27,28]. Apart from its laxative effect, ricinoleic acid causes intestinal dysfunction via local inflammation and stimulation of prostaglandin biosynthesis, which also inhibits reabsorption of ions and water [29]. In all antidiarrheal assays, loperamide was used as a standard drug, which enhances the rate of absorption by reducing the volume and movement of intestinal contents [30].

In castor oil-induced diarrhea, the ethanol extract of *O. rugosa* produced a remarkable inhibitory effect, in terms of both defecation rate and diarrhea. The extract, at all doses (100, 200, 400 mg/kg) decreased the total number of feces at 1h intervals over 4h, while diarrheal feces were reduced, indicating an alteration of defecation frequency and consistency. Among all three doses of EEOR, 200 and 400 mg/kg significantly (*P* < 0.001) reduced defecation numbers by 52.05% and 60.27% respectively, which indicates a dose-dependent antidiarrheal action. A dose of the extract with 400 mg/kg EEOR exhibited inhibition (62.50%) of diarrhea that was comparable to the standard drug loperamide (65.62%). This demonstrates that a relatively high dose of EEOR is required to evoke the desired response, and a similar phenomenon has been observed by similar studies on different plant species [31].

The anti-enteropooling potential of EEOR was investigated to explore its antidiarrheal efficacy further and to aid mechanistic interpretation. Our results show that the extract markedly inhibited castor oil-induced enteropooling into the small intestine, likely through suppressing castor oil stimulated prostaglandin biosynthesis. All tested doses significantly decreased intraluminal fluid compared to the control, with the highest dose of 400 mg/kg decreasing both the volume by 32.29% (*P* < 0.05) and weight of intestinal contents by 49.57% (*P* < 0.001). These results confirm the antidiarrheal efficiency of our extract and are comparable with an analogous study conducted by Agbon et al. [32].

To further characterize the effect of EEOR in reducing intestinal hypermotility, we investigated gastrointestinal motility using a charcoal meal tracer. We observed that the administration of the extract delayed the transit of the charcoal marker through the entire intestine. This inhibitory effect was seen with all doses employed and implies that an anti-motility action underlies the mechanism of action of the extract. Maximal inhibition of the peristaltic index was exhibited following a dose of 400 mg/kg (41.66%, *P* < 0.001), and was equipotent with the standard drug loperamide (42.26%, *P* < 0.001). Our findings suggest that the extract both decreases hypermotility and increases the transit time through the suppression of intestinal muscle spasm, thus extending the time for absorptive processes [33].

As aforementioned, castor oil promotes prostaglandin biosynthesis, which leads to the release of various pro-inflammatory mediators, leading to inflammation and irritation. Non-steroidal anti-inflammatory drugs (NSAIDs) may prevent diarrhea through inhibition of castor oil stimulated prostaglandin synthesis [34]. In this study, we assessed the anti-inflammatory activity of EEOR following histamine challenge. Histamine causes contraction of the smooth muscle of small intestine, uterus, bronchi, and bronchioles through activation of H1-receptors [35]. The mechanism of the local inflammatory response induced by histamine is through the activation of vasodilation, edema formation, vascular permeability, and cytokine release. [36]. Our results showed that EEOR significantly (*P* < 0.001) suppressed histamine-induced paw edema, which provides evidence of a potential anti-inflammatory effect. EEOR may thus ameliorate an acute inflammatory response via inhibition of prostaglandins or other inflammatory mediators.

In the anthelmintic study, we utilized the aquatic worm *Tubifex tubifex*, a species of aquatic oligochaete that is a suitable host for the *Myxobolus cerebralis* parasite, responsible for whirling disease in salmonid fish [37]. Our data revealed that exposure to EEOR dose-dependently reduced (*P* < 0.001) both paralysis and death times of the worm, indicating the presence of a potential anthelmintic compound(s). The reference drug levamisole (a nicotinic receptor agonist) activates excitatory nicotinic acetylcholine (nACh) receptors on the muscle of the worm, causing paralysis and death [38], and a similar mechanism may account for the anthelmintic action of EEOR.

We investigated the antimicrobial activity of EEOR through the disc diffusion method, and the extract induced a significant zone of inhibition against both *Bacillus subtilis* (a model Gram-positive microorganism) and *Escherichia coli* (Gram-negative microorganism) at concentrations of 500, 800 and 1000 μg/disc. The lowest concentration (500 μg/disc) failed to show activity against *Salmonella typhi* (Gram-negative microorganism), but the other two concentrations exhibited significant antibacterial activity. These results indicate the existence of a broad-spectrum antibiotic effect of the plant extract and represent the first such data on the extract. On the other hand, we did not find any noticeable effect of our extracts on the Gram-positive *Staphylococcus aureus* or *Bacillus cereus*, or on the Gram-negative organisms *Salmonella paratyphi* or *Pseudomonas aeruginosa*, even at 1000 μg/disc. Broadly, our results suggest that EEOR constituents may interrupt general cellular functions or disrupt bacterial membrane potential [39,40].

Generally, plants are rich in secondary metabolites with diverse biological actions, acting as natural defense mechanisms against bacteria, insects, viruses, and fungi. Our preliminary phytochemical evaluation suggested a distinct phytoconstituent profile in EEOR. Among these, alkaloids, flavonoids, phenols, tannins, terpenoids, and saponins are commonly reported to possess both antibacterial and anthelmintic activities [41,42]. Reports on various plant extracts suggest that antidiarrheal effects may also be mediated through the action of saponins, tannins, steroids flavonoids and alkaloids [43], whereas tannins and flavonoids are well known to aid reabsorption of intestinal fluids and electrolytes [44]. Additionally, tannins reduce intestinal motility by inhibiting bowel irritation, thereby exhibiting an antidiarrheal effect [45]. Various phytochemicals including flavonoids, steroids, and phenols have been ascribed anti-inflammatory actions [46]. As EEOR showed significant anthelmintic and antibacterial activity, especially on certain entero-pathogenic (*Bacillus subtilis*, *Salmonella typhi* and *Escherichia coli*) organisms, coupled with its observed effects on gut motility, this supports its possible utility in infectious diarrhea.

GC-MS analysis of EEOR identified a total of thirty different compounds. Based on the literature, thirteen of these have already been documented to be bioactive. Loliolide [47], ethyl linoleate [48], 2-palmitoylglycerol, and erucamide [49] have been shown to possess antibacterial activity, while γ-sitosterol, stigmasterol, vitamin E, and squalene [47] have both antibacterial and anti-inflammatory activities. Phytol and methyl palmitate have nematicidal, pesticidal, antibacterial, and anti-inflammatory activities. Notably, phytol is very active against *Salmonella typhi* [49]. Finally, neophytadiene [50] and methyl linoleate [47] have demonstrated anti-inflammatory activity.

Molecular docking studies have been widely used for the prediction of ligand-target interactions and to obtain better insights into the biological activity of natural products. It also gives additional clues about possible mechanisms of action and binding modes inside the binding pocket of various enzymes [51]. In order to obtain better insight into the observed biological activity (antidiarrheal, anthelmintic, and antibacterial) of EEOR constituents, thirteen representative compounds within EEOR were selected for docking analyses. These compounds were then docked against four targets, namely the M3 muscarinic acetylcholine receptor (PDB ID: 4U14), the 5-HT3 receptor (PDB ID: 5AIN), tubulin (PDB ID: 1SA0) and GlcN-6-P synthase (PDB: 1XFF).

Molecular docking studies with the M3 muscarinic acetylcholine receptor (PDB ID: 4U14) revealed that, among the thirteen compounds, seven interacted with several amino acid residues through hydrogen bonds and π-π stacking interactions (Tyr529, Tyr533, Ile222, Leu225, Asn152, Ser151, Tyr148), with docking scores ranging between −2.00 and −8.80 kcal/mol. On the other hand, five compounds interacted with a number of amino acid residues (Ile116, Glu191, Arg57, Arg57, and Thr34) within the 5-HT3 receptor (PDB ID: 5AIN) with docking scores ranging from −0.69 to −5.47 kcal/mol. From these results, we can conclude that the studied phytoconstituents may in part be responsible for the antidiarrheal activity of EEOR through interaction with these target proteins.

In the anthelmintic docking study, the thirteen compounds were docked with tubulin (PDB ID: 1SA0) and showed docking scores ranging from −0.59 to −7.13 kcal/mol. From the results, it is clear that the phytoconstituent stigmasterol displayed the highest score against tubulin, followed by γ-sitosterol, vitamin E, ethyl linolenate, loliolide, erucamide, phytol, methyl linoleate, methyl palmitate, and neophytadiene. It has been previously reported that phytol and methyl palmitate possess nematicidal and pesticidal activities [49], and the anthelmintic activity of EEOR may be related to these phytoconstituents. In the case of the antibacterial docking study, loliolide had the highest binding affinity towards the GlcN-6-P synthase enzyme (PDB: 1XFF), followed by ethyl linolenate, erucamide, 2-palmitoylglycerol, phytol, methyl linoleate, neophytadiene, methyl palmitate, and methyl stearate. The antibacterial activity of the EEOR may thus be explained by the presence of loliolide, ethyl linolenate, erucamide, 2-palmitoylglycerol, and phytol, which have good docking scores and for which bioactivity has previously been reported [47,48].

All bioactive compounds were further characterized using the online-based prediction program ADME analysis to explore their drug-likeness, pharmacokinetics and physiochemical characteristics. Almost all compounds, except for γ -sitosterol, squalene, stigmasterol and vitamin E exhibited orally active drug-likeness properties, according to Lipinski’s rule. It is reported that compounds with lower molecular weight, lipophilicity, and hydrogen bond capacity have high permeability [52], good absorption and bioavailability [53,54]. However, this analysis does not assess if a compound has any particular pharmacological effect.

To predict a likely pharmacological profile of the compounds, we utilized the structure-based biological activity prediction program Prediction of Activity Spectra for Substances (PASS). The results suggested several activities, among these, we established probable activity values (Pa range 0.235–0.826) for all 13 compounds for anthelmintic, antibacterial, anti-inflammatory, spasmolytic and antiprotozoal actions, supporting our laboratory investigations of EEOR. Moreover, other activities were predicted, suggesting the broader potential of this species. In summary, our comprehensive analyses, utilizing complementary tools, support the traditional uses of EEOR. The observed effects may be due to the combined actions of several phytoconstituents, both those documented herein and potentially other as yet uncharacterized compounds.

## 4. Materials and Methods

### 4.1. Drugs and Chemicals

All drugs and chemicals used in this research were of analytical grade. Loperamide was obtained from Square Pharmaceuticals Ltd. (Dhaka, Bangladesh), levamisole from ACI Limited (Dhaka, Bangladesh), and castor oil from WELL’s Health Care (Madrid, Spain). Ethanol (Merck, Darmstadt, Germany), Kanamycin (Sigma Chemical Co., St. Louis, MO, USA) and histamine (BDH Chemicals Ltd. Poole, UK) were procured from the mentioned sources.

### 4.2. Chemical Compounds Studied in This Article

Loliolide (PubChem CID: 100332); Ethyl linolenate (PubChem CID: 6371716); Methyl linoleate (PubChem CID: 5284421); Erucamide (PubChem CID: 5365371); γ-Sitosterol (PubChem CID: 457801); 2-Palmitoylglycerol (PubChem CID: 123409); Methyl Palmitate (PubChem CID: 8181); Methyl stearate (PubChem CID: 8201); Neophytadiene (PubChem CID: 10446); Phytol (PubChem CID: 5280435); Squalene (PubChem CID: 638072); Stigmasterol (PubChem CID: 5280794); Vitamin E (PubChem CID: 14985).

### 4.3. Plant Collection, Identification, and Extraction

The leaves of *Ophiorrhiza rugosa* var. *prostrata* (D.Don) Deb & Mondal were collected in September 2017 from Kaptai National Park (22°30′08″N 92°12′04″E), Rangamati District, Chittagong Division, Bangladesh. The plant was certified and authenticated by Dr. Shaikh Bokhtear Uddin, Professor, Ethno-botany and Pharmacognosy Lab, Department of Botany, University of Chittagong, Bangladesh, with a voucher specimen (accession no: 7609 CTGUH) deposited in the Herbarium of the University of Chittagong (CTGUH). After subsequent washing with normal and distilled water, the collected leaves were cut and oven-dried for a week at constant temperature (50 °C), before milling into a coarse powder using an automatic grinder. Then, the fine powder (350 g) was soaked in 850 mL of ethanol for seven days at room temperature, with regular shaking and stirring on a shaker machine (model VTRS-1, Nunes Instruments, Tamil Nadu, India). After 7 days, the macerate was filtered through a sterilized cotton plug followed by Whatman filter paper No. 1, and the eluting solvent evaporated on a rotary evaporator (RE 200, Sterling, Norman Way Industrial Estate, Cambridge, UK) at room temperature to afford a semisolid extract (EEOR: 10 g), which was kept in a refrigerator (−4 °C) until further use.

### 4.4. Animals and Ethical Statements

Adult Swiss albino mice (20–25 g) of both sexes were obtained from Jahangir Nagar University, Savar, Dhaka, Bangladesh. The animals were housed in polypropylene cages for adaptation, under standard laboratory conditions (room temperature 25 ± 2 °C; relative humidity 55–60%, 12 h light/dark cycle), with food pellets and water *ad libitum*. All animals were acclimatized for 2 weeks and fasted overnight before starting all experiments. This experiment was designed based on the Ethical Principles and Guidelines guided by The Swiss Academy of Medical Sciences and the Swiss Academy of Sciences. All tests were run in a remote and noiseless ambiance, between 9.00 a.m. and 5.00 p.m. The study protocol was approved by both the Ethical review committee and the P&D committee of the Department of Pharmacy, International Islamic University Chittagong, Bangladesh under the code Pharm-P&D-61/08′16-122.

### 4.5. GC-MS (Gas Chromatography-Mass Spectroscopy) Analysis of EEOR 

GC-MS analysis of EEOR was evaluated using a model 7890A capillary gas chromatograph along with a mass spectrometer (Agilent Technologies, Santa Clara, CA, USA). The column was a fused silica capillary column of 95% dimethyl-poly-siloxane and 5% phenyl (HP-5MSI; length: 90 m, diameter: 0.250 mm and film: 0.25 µm). Parameters for GC-MS detection were an injector temperature of 250 °C, an initial oven temperature of 90 °C gradually raised to 200 °C at a speed of 3 °C/min for 2 min and with a final increase to 280 °C at 15 °C/min for 2 min. The total GC-MS run time was 36 min, using 99.999% helium as a carrier gas, at a column flow rate of 1 mL/min. The GC to MS interface temperature was fixed at 280 °C, and an electron ionization system was set on the MS in scan mode. The mass range evaluated was 50–550 *m*/*z*, where MS quad and source temperatures were maintained at 150 °C and 230 °C respectively. The NIST-MS Library 2009 was used to search and identify each component, and to measure the relative percentage of each compound, relative peak areas of the TIC (total ionic chromatogram) were used, with calculations performed automatically.

### 4.6. Acute Toxicity Testing of EEOR

Acute toxicity testing was assessed under standard laboratory conditions following OECD guidelines [55]. Animals (*n* = 6) of the control and test groups were each administered 1% Tween-80 or a single oral dose (5, 50, 100, 200, 400, 1000 and 2000 mg/kg body weight) of the test extract (EEOR). Before administration of extract, mice were kept fasting overnight, and food was also delayed for 3 to 4 h after receiving the extract. All experimental animals were observed individually, paying particular attention to any unexpected responses, including behavioral changes, allergic syndromes (itching, skin rash), and mortality over the next 72 h.

### 4.7. Qualitative Phytochemical Screening of EEOR

Qualitative phytochemical analysis of EEOR was carried out following standard procedures, as previously reported by Tiwari et al. [56].

### 4.8. Antidiarrheal Activity Evaluation of EEOR (In Vivo)

#### 4.8.1. Castor Oil-Induced Diarrhea

The conditions of Awouters et al. [34] were followed with slight modifications. Mice were fasted overnight prior to the experiment. Experimental animals were separated randomly into control and test groups consisting of 6 mice in each category. Group-I served as a negative control, and received 1% Tween-80 in distilled water; group-II (positive control) received loperamide (5 mg/kg BW; p.o), while test groups III-V were treated with EEOR (100, 200 and 400mg/kg BW; p.o) respectively. After 1 h, each mouse was put into an individual cage and diarrhea induced (0.5 mL castor oil, p.o). Blotting paper on the floor of each cage was monitored to observe both the number and consistency of fecal droppings. Blotting papers were replaced every 60 min during the 4h observation period. The total numbers of both dry and wet feces excreted by the animals were counted. The following equation was used to calculate percent inhibition of diarrhea:

(1) $$\mathrm{percentage}\;\mathrm{of}\;\mathrm{inhibition}\;\mathrm{of}\;\mathrm{diarrhea}=\frac{\mathrm{Total}\;\mathrm{number}\;\mathrm{of}\;\mathrm{diarrheal}\;\mathrm{faces}\times \left(\mathrm{control}-\mathrm{test}\;\mathrm{groups}\right)\;}{\mathrm{Total}\;\mathrm{number}\;\mathrm{of}\;\mathrm{diarrheal}\;\mathrm{faces}\;\mathrm{of}\;\mathrm{the}\;\mathrm{control}}\times 100,$$

#### 4.8.2. Castor Oil-Induced Enteropooling

Intraluminal fluid accumulation was evaluated by the method described by Robert et al. [57]. Dosing treatments were as for castor oil-induced diarrheal testing, again with six animals per group. One hour post administrations of each test dose, animals were treated with castor oil (0.5 mL) to induce diarrhea. Two hours later, the mice were sacrificed, and the small intestine was isolated from pyloric sphincter to caecum. The small intestine was weighed (g) and the volume of intestinal contents (ml) was measured by milking into a graduated tube. The intestine was reweighed, and the differences between full and empty intestines were calculated. To calculate, the percentage volume and weight of intestinal contents were determined using the following formula:

(2) $$\mathrm{percentage}\;\mathrm{of}\;\mathrm{inhibition}=\frac{\mathrm{Mean}\;\mathrm{of}\;\mathrm{intestinal}\;\mathrm{content}\times \left(\mathrm{control}-\mathrm{test}\;\mathrm{groups}\right)}{\mathrm{Mean}\;\mathrm{of}\;\mathrm{intestinal}\;\mathrm{content}\;\mathrm{of}\;\mathrm{the}\;\mathrm{control}}\times 100,$$

#### 4.8.3. Gastrointestinal Motility

This experiment was performed based on the method of Mascolo et al. [58], with the treatment of animals of each group (*n* = 6) as described in the castor oil-induced diarrhea test. In brief, 0.5 mL of castor oil was administered to each animal to induce diarrhea. One hour after administration of each test dose, animals were treated orally with 1 mL of a charcoal meal (10% charcoal suspension in 5% gum acacia). After 1 h, animals were sacrificed and the distance traveled by the charcoal meal from the pylorus to caecum was measured (cm) and expressed as a percentage of the total distance of the intestine. The following formulae were used to express the percentage of inhibition and Peristalsis index:

(3) $$\mathrm{inhibition}\;(\%)=\frac{\mathrm{Distance}\;\left(\mathrm{cm}\right)\;\mathrm{travel}\;\mathrm{by}\;\mathrm{the}\;\mathrm{charcoal}\times \left(\mathrm{control}-\mathrm{test}\;\mathrm{groups}\right)}{\mathrm{Distance}\;\mathrm{travel}\;\mathrm{by}\;\mathrm{the}\;\mathrm{charcoal}\;\mathrm{in}\;\mathrm{the}\;\mathrm{control}\;\mathrm{group}}\times 100,$$

(4) $$\mathrm{Peristalsis}\;\mathrm{Index}=\frac{\mathrm{Distance}\;\mathrm{travel}\;\mathrm{by}\;\mathrm{the}\;\mathrm{charcoal}\;\mathrm{meal}}{\mathrm{Total}\;\mathrm{length}\;\mathrm{of}\;\mathrm{the}\;\mathrm{small}\;\mathrm{intestine}}\times 100,$$

### 4.9. Histamine-Induced Paw Edema

The anti-inflammatory activity of EEOR was evaluated following injection of histamine into the plantar surface of the mouse hind paw [59]. Animals were divided into four groups (*n* = 6); Group I (negative control) received 1% Tween-80 (2 mL/kg); Group II (Positive control) received diclofenac sodium (10 mg/kg BW; p.o); and Groups III and IV received EEOR (200 and 400 mg/kg BW; p.o) respectively. 30 min following treatment, 0.05 mL histamine (1 mg/kg, in 1% Tween-80 with D.W) was injected in the sub-plantar area of the right paw of each mouse to induce acute inflammation, and micrometer slide calipers were used to measure the paw volume at 1, 2, 3 and 4 h. The percentage inhibition of the inflammatory effect of the extract was calculated using the following expression:

(5) $$\%\;\mathrm{inhibition}\;\mathrm{of}\;\mathrm{inflammation}=\frac{\mathrm{Mean}\;\mathrm{degree}\;\mathrm{of}\;\mathrm{inflammation}\;\left(\mathrm{control}-\mathrm{test}\;\mathrm{groups}\right)}{\mathrm{Mean}\;\mathrm{degree}\;\mathrm{of}\;\mathrm{inflammation}\;\mathrm{of}\;\mathrm{control}}\times 100$$

### 4.10. Anthelmintic Activity of EEOR (In Vitro)

Anthelmintic activity was assessed following the method of Ajaiyeoba et al. with slight modifications [60,61]. In this experiment, the sludge worm, or sewage worm (*Tubifex tubifex*, size: 2 to 2.5 cm in length), was used for its physiological and anatomical relevance to intestinal worms, e.g., Annelida. Testing was performed in triplicate. In brief, 5 to 10 worms were randomly placed in each Petri dish, divided into four groups (I, II, III and IV). To each, 3 mL of either EEOR at a specified concentration (5, 8 or 10 mg/mL) or the standard drug levamisole (1 mg/mL) added. Anthelmintic activity was calculated at two different stages, namely ‘time of paralysis’ and ‘time of death’ of the worms. Time to paralysis was counted as the time when worms lost their natural movement. The time of death was recorded after confirming that the worms moved neither when vigorously shaken nor when dipped in slightly warm water.

### 4.11. Antibacterial Activity of EEOR (In Vitro)

The antibacterial effect of EEOR was evaluated by the disc diffusion technique [62]. Prepared Nutrient agar was placed into Petri dishes under laminar airflow for solidification. Overnight cultures of Gram-positive *Bacillus subtilis* (ATCC 6633), *Staphylococcus aureus* (ATCC 6538) and *Bacillus cereus* (ATCC 14579) and Gram-negative *Salmonella typhi* (ATCC 29629), *Salmonella paratyphi* (ATCC 9150), *Escherichia coli* (ATCC 8739) and *Pseudomonas aeruginosa* (ATCC 9027) organisms were each prepared with 100 µL bacteria (bacterial inocula were adjusted to 10^8^ CFU/mL), spread smoothly on the agar surface. Dry sterile discs (6mm diameter) were laid upon the seeded agar plate using a sterile forceps. Each desired concentration of EEOR (500, 800 or 1000 μg) was loaded on these discs and then incubated (at 37 °C for 24 h). The diameter of each zone of inhibition was recorded and measured in mm. As a positive control, kanamycin (30 μg/disc) was used.

### 4.12. In silico Molecular Docking

The major bioactive compounds of EEOR, as detected by GC-MS, were selected for molecular docking studies, to understand better possible molecular interactions based on their affinity to interact with different target proteins. Docking studies were performed using the Schrödinger suite-Maestro v10.1, LLC, New York, NY, USA, and Accelrys Discovery Studio 4.0 software (BIOVIA, San Diego, CA, USA) was used for visualization of 3D structures.

#### 4.12.1. Ligand Preparation

The structures of thirteen major compounds were obtained from the PubChem compound repository, and the ligands prepared using the LigPrep tool embedded in Maestro v 10.1 (Schrödinger suite, LLC New York, NY, USA), neutralized at pH 7.0 ± 2.0 using Epik 2.2, and minimized by force field OPLS_2005.

#### 4.12.2. Receptor Preparation

3D crystal structures of the proteins used for the test were downloaded from the Protein Data Bank; RCSB PDB [63], GlcN-6-P synthase (PDB ID: 1XFF) [64], tubulin (PDB ID: 1SA0) [65] 5-HT3 receptor (PDB ID: 5AIN) [66] and M3 muscarinic acetylcholine receptor (PDB ID: 4U14) [67]. The Protein Preparation Wizard of the Schrödinger suite-Maestro version 10.1 was used to prepare and refine the crystal structures. Charges and bond orders were assigned, hydrogens added to heavy atoms and selenomethionines and selenocysteines converted into methionines and cysteines respectively, followed by removing all water molecules. Using force field OPLS_2005, minimization was performed to set a maximum heavy atom RMSD to 0.30 Å.

#### 4.12.3. Grid Generation and Molecular Docking

Receptor grid generation and molecular docking experiments were performed using Glide (Schrödinger suite-Maestro version 10.1) [68,69] For each protein, a grid was produced using the following default parameters: van der Waals scaling factor 1.00 and charge cut-off value 0.25, subjected to the OPLS_2005 force field. A cubic box of definite dimensions centered on the centroid of the active site residues was generated for the receptor, and the box size was set to 14 Å × 14 Å × 14 Å for docking. Docking experiments were carried out using the Standard Precision (SP) scoring function of Glide, and only the best scoring fit with docking score was noted for each ligand.

### 4.13. In Silico ADME Analysis

The pharmacokinetic properties of all major identified compounds were evaluated using Lipinski’s rule of five [70]. Lipinski stated that a compound could show drug-like behavior if it does not fail more than one of the following criteria: (i) molecular weight not more than 500; (ii) H-bond donors ≤5; (iii) H-bond acceptors ≤10; (iv) Lipophilicity <5; and (v) molar refractivity between 40 and 130. The web tool Swiss ADME [71] was used to assess the ADME parameters of all compounds. Compounds which obey Lipinski rule are considered as ideal drug candidates.

### 4.14. In Silico PASS Prediction

Possible biological activities of identified major compounds were evaluated using the online computer program PASS (Prediction of Activity Spectra for Substances) [72]. This tool predicts up to 3750 biological properties of a compound, associated with an analysis of its chemical structure. The outcomes of this analysis were denoted as Pa (probable activity) and Pi (probable inactivity), where the values of both Pa and Pi may differ from 0.000 to 1.000. We considered values of P_a_ > P_i_ and Pa > 0.700 to indicate biological activity for a compound [73].

## 5. Statistical Analysis

Data were analyzed using SPSS 20.0 statistical software (SPSS, IBM Corporation, Armonk, NY, USA). Results were presented as mean ± SEM (standard error of the mean), and one-way ANOVA followed by Dunnett’s test was applied. A *p*-value of less than 0.05 was considered significant.

## 6. Conclusions

In summary, our study demonstrates that EEOR possesses significant and dose-dependent antidiarrheal activity in different models, which supports the traditional use of this plant in folk medicine. The study also provides further evidence of inhibition of inflammatory mediators, which rationalise the anti-inflammatory activity of the plant extract. The positive results regarding anthelmintic and antibacterial activities increase the value of this plant. Collectively, these outcomes support the ethnomedicinal use of *O. rugosa* for the management of various infectious diseases. Furthermore, various potential bioactive constituents identified by GC-MS analysis showed promising binding affinity toward different proteins in molecular docking experiments, and their drug-like characteristics were demonstrated through ADME/T analysis. PASS predictions of bioactive constituents were in agreement with our laboratory findings. Therefore, *O. rugosa* may represent a viable candidate for the treatment of infectious diseases. However, further studies are needed to identify and isolate the pure compounds responsible for the observed biological effects, and to characterize its toxicity profile and longer-term safety.

## Figures and Tables

**Table 1:** List of compounds identified in EEOR by GC-MS analysis.

S.N.	RT (min)	PA (%)	Name of Compound	Molecular Formula
1	7.624	0.92	Carbonic acid, hexadecyl methyl ester	C_18_H_36_O_3_
2	10.130	1.77	1-Nonadecene	C_19_H_38_
3	12.235	1.29	Succinic acid, tridec-2-yn-1-yl *trans*-4-methylcyclohexyl ester	C_24_H_40_O_4_
4	12.388	1.10	6-Hydroxy-4,4,7α-trimethyl-5,6,7,7α-tetrahydrobenzofuran-2(4*H*)-one, or Loliolide	C_11_H_16_O_3_
5	12.471	1.97	1-Nonadecene	C_19_H_38_
6	12.585	1.38	2-Cyclohexen-1-one, 4-hydroxy-3,5,6-trimethyl-4-(3-oxo-1-butenyl)-	C_13_H_18_O_3_
7	13.003	0.86	Neophytadiene	C_20_H_38_
8	13.918	3.95	Hexadecanoic acid, methyl ester or Methyl Palmitate	C_17_H_34_O_2_
9	14.588	5.47	9H-Pyrido[3,4-b]indole, 1-methyl-	C_12_H_10_N_2_
10	15.659	2.96	9,12-Octadecadienoic acid (*Z*,*Z*)-, methyl ester or Methyl linoleate	C_19_H_34_O_2_
11	15.716	4.33	8,11,14-Docosatrienoic acid, methyl ester	C_23_H_40_O_2_
12	15.843	15.50	Phytol	C_20_H_40_O
13	15.939	1.21	Methyl stearate	C_19_H_38_O_2_
14	16.366	1.17	9,12,15-Octadecatrienoic acid, ethyl ester, (*Z*,*Z*,*Z*)- or Ethyl linolenate	C_20_H_34_O_2_
15	17.203	1.92	Octadecanoic acid, 3-hydroxypropyl ester	C_21_H_42_O_3_
16	17.825	1.61	1-Heptatriacotanol	C_37_H_76_O
17	18.823	2.21	6,9-Octadecadienoic acid, methyl ester	C_19_H_34_O_2_
18	19.377	0.91	Hexadecanoic acid, 2-hydroxy-1-(hydroxy- methyl)ethyl ester, or 2-Palmitoylglycerol	C_19_H_38_O_4_
19	19.726	0.95	Diisooctyl phthalate	C_24_H_38_O_4_
20	20.844	2.87	*E*,*E*,*Z*-1,3,12-Nonadecatriene-5,14-diol	C_19_H_34_O_2_
21	20.914	1.10	Ethyl 9,12,15-octadecatrienoate	C_20_H_34_O_2_
22	21.522	5.39	13-Docosenamide, (*Z*)- or Erucamide	C_22_H_43_NO
23	21.885	4.83	Squalene	C_30_H_50_
24	22.372	1.14	α-Tocospiro B	C_29_H_50_O_4_
25	22.765	0.92	1,6,10,14,18,22-Tetracosahexaen-3-ol, 2,6,10,15,19,23-hexamethyl-, (all-*E*)-	C_30_H_50_O
26	24.931	2.51	Vitamin E	C_29_H_50_O_2_
27	26.431	5.00	Campesterol	C_28_H_48_O
28	26.876	7.92	Stigmasterol	C_29_H_48_O
29	27.768	14.94	γ-Sitosterol	C_29_H_50_O
30	31.229	1.91	Lup-20(29)-en-3-ol, acetate, (3β)-	C_32_H_52_O_2_

RT: Retention time; PA: Peak area.

**Table 2:** The effect of *Ophiorrhiza rugosa* extract on feces count in castor oil-induced diarrhea in mice.

Treatment (mg/kg)	Total Number of Feces	% Inhibition of Defecation	Total Number of Diarrheal Feces	% Inhibition of Diarrhea
Control (0.1 mL/mouse)	14.60 ± 0.74		6.40 ± 0.81	
Loperamide (5)	5.40 ± 0.24 ***	63.01	2.20 ± 0.20 ***	65.62
EEOR (100)	8.00 ± 0.44 **	45.20	5.00 ± 0.31 ***	21.87
EEOR (200)	7.00 ± 0.83 ***	52.05	3.80 ± 0.48 **	40.62
EEOR (400)	5.80 ± 0.20 ***	60.27	2.40 ± 0.24 ***	62.50

Significantly different when compared with that of the control group at ** *P* < 0.01, *** *P* < 0.001. Results are presented as mean ± SEM (*n* = 6).

**Table 3:** The effect of *Ophiorrhiza rugosa* extract on castor oil-induced enteropooling in mice.

Treatment (mg/kg)	Volume of Intestinal Content (mL)	% Inhibition	Weight of Intestinal Content (gm)	% Inhibition
Control (0.1 mL/mouse)	0.51 ± 0.025		0.71±0.022	
Loperamide (5)	0.26 ± 0.013 ***	49.42	0.29±0.012 ***	58.87
EEOR (100)	0.44 ± 0.014 **	13.22	0.55±0.030 **	22.25
EEOR (200)	0.40 ± 0.017 ***	21.78	0.44±0.090 **	38.02
EEOR (400)	0.34 ± 0.063 *	32.29	0.35±0.047 ***	49.57

Significantly different when compared with that of the control group at * *P* < 0.05, ** *P* < 0.01, *** *P* < 0.001. Results are presented as mean ± SEM (*n* = 6).

**Table 4:** The effect of *Ophiorrhiza rugosa* extracts on intestinal transit in mice using a charcoal meal as a marker.

Treatment (mg/kg)	Total Length of Intestine (cm)	Distance Travelled by Marker (cm)	Peristalsis Index (%)	% Inhibition Relative to Control
Control (0.1 mL/mouse)	48.60 ± 0.51	41.40 ± 0.93	85.19 ± 1.74	
Loperamide (5)	49.20 ± 0.58	20.80 ± 0.73	42.26 ± 1.32 ***	57.73
EEOR (100)	44.20 ± 0.37 **	29.20 ± 0.58 **	66.07 ± 1.32 ***	33.92
EEOR (200)	43.00 ± 0.44 **	24.80 ± 0.86 ***	57.69 ± 2.08 ***	42.30
EEOR (400)	48.30 ± 0.25 ***	20.10 ± 1.36 ***	41.66 ± 3.02 ***	58.33

Significantly different when compared with that of the control group at ** *P* < 0.01, *** *P* < 0.001. Results are presented as mean ± SEM (*n* = 6).

**Table 5:** Anti-inflammatory activity of *Ophiorrhiza rugosa* extract on histamine-induced paw edema.

Treatment (mg/kg)	Paw Volume (mm) (% Inhibition)			
	1 h	2 h	3 h	4 h
Control (0.1mL/mouse)	0.454 ± 0.010	0.392 ± 0.012	0.340 ± 0.007	0.312 ± 0.008
Diclofenac-Na (10)	0.350 ± 0.004 *** (42.42)	0.290 ± 0.007 *** (60.29)	0.264 ± 0.010 *** (66.66)	0.248 ± 0.012 *** (78.57)
EEOR (100)	0.422 ± 0.005 ** (11.11)	0.358 ± 0.015 *** (17.64)	0.310 ± 0.010 *** (23.8)	0.290 ± 0.004 ** (21.42)
EEOR (200)	0.398 ± 0.007 *** (20.20)	0.334 ± 0.009 *** (30.88)	0.294 ± 0.006 *** (35.71)	0.278 ± 0.006 *** (32.14)
EEOR (400)	0.344 ± 0.012 *** (38.38)	0.300 ± 0.006 *** (42.64)	0.260 ± 0.005 *** (54.76)	0.246 ± 0.005 *** (57.14)

Each value is expressed as mean ± SEM (*n* = 6). ** *P* < 0.01, *** *P* < 0.001 compared with the control group (Dunnett’s test).

**Table 6:** Antibacterial effects of the ethanol extract of *Ophiorrhiza rugosa* leaves.

Bacterial Strain	Name of the Bacteria	Zone of Inhibition (mm)			
		Concentration (μg/disc)			Kanamycin (30 µg/disc)
		EEOR 500	EEOR 800	EEOR 1000	
Gram-positive	*Staphylococcus aureus* (ATCC 6538)	-	-	-	29.30 ± 0.60
	*Bacillus subtilis* (ATCC 6633)	7.33 ± 0.57	11.70 ± 0.75	14.80 ± 0.72	32.81 ± 0.67
	*Bacillus cereus* (ATCC 14579)	-	-	-	27.50 ± 0.58
Gram-negative	*Salmonella typhi* (ATCC 29629)	-	7.33 ± 0.57	12.80 ± 0.34	28.218±0.81
	*Salmonella paratyphi* (ATCC 9150)	-	-	-	30.51 ± 0.50
	*Escherichia coli* (ATCC 8739)	8.20 ± 0.72	11.26 ± 1.16	16.23 ± 0.68	31.20 ± 0.82
	*Pseudomonas aeruginosa* (ATCC 9027)	-	-	-	26.28 ± 0.36

Values are presented as mean inhibition zone (mm) ± SD of three replicates; -: no activity.

**Table 7:** Docking scores of the major bioactive compounds.

Compound Name	Docking Score ^1^			
	4U14	5AIN	1SA0	1XFF
Loliolide	−6.63	**−5.47**	−4.49	**−4.88**
Ethyl linolenate	−6.76	−3.47	−5.36	−3.10
Methyl linoleate	−3.26	−1.65	−1.87	0.25
Erucamide	−	−	−2.35	−1.21
γ-Sitosterol	−	−	−7.00	−
2-Palmitoylglycerol	−3.55	−	−	−1.16
Methyl palmitate	−2.00	−0.25	−1.10	+1.81
Methyl stearate	−	+1.62	−	+2.76
Neophytadiene	−2.55	−0.69	−0.59	+1.18
Phytol	−3.62	−2.08	−2.30	−0.12
Squalene	−	−	−	−
Stigmasterol	−	−	**−7.13**	−
Vitamin E	**−8.80**	−	−6.65	−
Reference drugs (Loperamide/Levamisole/Kanamycin)	−7.32	−	−6.26	−2.73

^1^ Docking scores in kcal/mol; Bold text indicates the highest score.

**Table 8:** ADME property prediction for the major compounds of EEOR, obtained using Swiss ADME.

Compound Name	MW ^1^ (g/mol)	HB Acceptor ^2^	HB Donor ^3^	Log P_o/w_^4^	Molar Refractivity ^5^	Rule of Five ^6^
Loliolide	196.24	3	1	1.53	52.51	0
Ethyl linolenate	306.48	2	0	5.82	98.12	1
Methyl linoleate	297.47	2	0	5.69	98.78	1
Erucamide	337.58	1	1	6.77	110.30	1
γ-Sitosterol	414.71	1	1	7.19	133.1	2
2-Palmitoylglycerol	330.50	4	2	4.72	97.06	0
Methyl palmitate	270.45	2	0	5.54	85.12	1
Methyl stearate	298.50	2	0	6.24	94.73	1
Neophytadiene	278.52	0	0	7.07	97.31	1
Phytol	296.53	1	1	6.22	98.94	1
Squalene	410.72	0	0	9.38	143.48	2
Stigmasterol	412.69	1	1	6.96	132.75	2
Vitamin E	430.71	2	1	8.27	139.27	2

^1^ MW, Molecular weight (acceptable range: <500). ^2^ HB, Hydrogen bond acceptor (acceptable range: ≤10). ^3^ HB, Hydrogen bond donor (acceptable range: ≤5). ^4^ Lipophilicity (expressed as Log P_o/w_, acceptable range: <5). ^5^ Molar refractivity should be between 40 and 130. ^6^ Rule of five: Number of violations of Lipinski’s rule of five; recommended range: 0–4.

**Table 9:** Biological activities predicted for *Ophiorrhiza rugosa* major compounds by PASS online.

Compound Name	Biological Properties Predicted by Pass Online	Pa	Pi
Loliolide	Sugar-phosphatase inhibitor	0.727	0.028
	Antibacterial	0.418	0.026
	Spasmolytic, urinary	0.454	0.062
	Anti-inflammatory	0.416	0.088
	Antiperistaltic	0.345	0.018
	Antihelmintic	0.345	0.071
Ethyl linolenate	Lipid metabolism regulator	0.951	0.003
	Anti-inflammatory	0.826	0.005
	Histamine release inhibitor	0.523	0.028
	Antiparasitic	0.489	0.017
	Antihelmintic	0.488	0.019
	Anti-inflammatory, intestinal	0.438	0.015
Methyl linoleate	Lipid metabolism regulator	0.881	0.004
	Antisecretoric	0.781	0.005
	Anti-inflammatory	0.727	0.013
	Reductant	0.637	0.009
	Antihelmintic (Nematodes)	0.500	0.017
	Anti-infective	0.424	0.038
Erucamide	Sugar-phosphatase inhibitor	0.828	0.012
	Anti-infective	0.501	0.022
	Prostaglandin E1 antagonist	0.470	0.005
	Anti-inflammatory, intestinal	0.444	0.014
	Albendazole monooxygenase inhibitor	0.450	0.026
	Antitoxic	0.387	0.025
γ-Sitosterol	Antihypercholesterolemic	0.977	0.001
	Antiviral (Influenza)	0.686	0.006
	Antiinflammatory	0.572	0.038
	Antiacne	0.529	0.005
	Antiprotozoal (*Leishmania*)	0.316	0.091
	Antibacterial	0.282	0.067
2-Palmitoylglycerol	Sugar-phosphatase inhibitor	0.927	0.003
	Lipid metabolism regulator	0.889	0.004
	Antiinfective	0.757	0.005
	Anti-inflammatory, intestinal	0.578	0.004
	Histamine release inhibitor	0.573	0.015
	Antiprotozoal (*Leishmania*)	0.560	0.018
Methyl palmitate	Anti-inflammatory, intestinal	0.758	0.002
	Calcium channel (voltage-sensitive) activator	0.637	0.014
	Antihelmintic (Nematodes)	0.619	0.005
	Reductant	0.523	0.020
	Antimutagenic	0.513	0.014
	Antiprotozoal (*Leishmania*)	0.442	0.035
Methyl stearate	GABA aminotransferase inhibitor	0.820	0.003
	Anti-inflammatory, intestinal	0.758	0.002
	Lipid metabolism regulator	0.740	0.009
	Gastrin inhibitor	0.716	0.004
	Antihelmintic (Nematodes)	0.619	0.005
	Antinociceptive	0.538	0.019
Neophytadiene	Carminative	0.691	0.007
	Gastrin inhibitor	0.641	0.012
	Antiulcerative	0.585	0.012
	Histamine release inhibitor	0.506	0.034
	Antiprotozoal (*Leishmania*)	0.460	0.031
	Antiparasitic	0.395	0.032
Phytol	Lipid metabolism regulator	0.828	0.005
	Antiparasitic	0.615	0.008
	Antihelmintic	0.605	0.004
	Antiprotozoal (*Leishmania*)	0.601	0.014
	Histamine release inhibitor	0.526	0.027
	Spasmolytic	0.506	0.027
Squalene	Sugar-phosphatase inhibitor	0.854	0.009
	Gastrin inhibitor	0.743	0.003
	Anti-inflammatory	0.699	0.016
	Antiparasitic	0.555	0.011
	Histamine release inhibitor	0.558	0.018
	Antihelmintic	0.538	0.005
Stigmasterol	Dermatologic	0.809	0.004
	Antiacne	0.552	0.004
	Antiinflammatory	0.541	0.045
	Antiprotozoal (*Leishmania*)	0.403	0.047
	Antisecretoric	0.367	0.068
	Bone formation stimulant	0.306	0.020
Vitamin E	Lipid peroxidase inhibitor	0.978	0.002
	Anti-inflammatory	0.830	0.005
	Free radical scavenger	0.783	0.003
	Spasmolytic	0.525	0.024
	Histamine release inhibitor	0.396	0.093
	Anti-infective	0.277	0.122

Pa = Probable activity; Pi = Probable inactivity.

## Supplementary Materials

The following are available online, Figure S1. 2D interactions of the best fit found for (A) Loliolide, (B) Ethyl linolenate, (C) Methyl linoleate, (D) 2-Palmitoylglycerol, (E) Methyl palmitate, (F) Neophytadiene, (G) Phytol, and (H) Vitamin E docked to the M3 muscarinic acetylcholine receptor (PDB ID: 4U14); Figure S2. Best ranked fit of (A) Loliolide, (B) Ethyl linolenate, (C) Methyl linoleate, (D) 2-Palmitoylglycerol, (E) Methyl palmitate, (F) Neophytadiene, (G) Phytol and (H) Vitamin E in the binding pocket of the M3 muscarinic acetylcholine receptor (PDB ID: 4U14); Figure S3. 2D interactions of the best fit found for (A) Loliolide, (B) Ethyl linolenate, (C) Methyl linoleate, (D) Methyl palmitate, (E) Methyl stearate and (F) Neophytadiene docked to the 5-HT3 receptor (PDB ID: 5AIN); Figure S4. Best ranked fit of (A) Loliolide, (B) Ethyl linolenate, (C) Methyl linoleate, (D) Methyl palmitate, (E) Methyl stearate and (F) Neophytadiene in the binding pocket of the 5-HT3 receptor (PDB ID: 5AIN); Figure S5. Best ranked fit of Phytol (A) in the binding pocket of 5-HT3 (PDB ID: 5AIN) and 2D representation of key interactions in the binding pocket for Phytol (B); Figure S6. 2D interactions of the best fit found for (A) Loliolide, (B) Ethyl linolenate, (C) Methyl linoleate, (D) Erucamide, (E) γ-Sitosterol, (F) Methyl palmitate, (G) Neophytadiene, (H) Phytol, (I) Stigmasterol and (J) Vitamin E docked to tubulin (PDB ID: 1SA0); Figure S7. Best ranked fit of (A) Loliolide, (B) Ethyl linolenate, (C) Methyl linoleate, (D) Erucamide, (E) γ-Sitosterol, (F) Methyl palmitate, (G) Neophytadiene, (H) Phytol, (I) Stigmasterol and (J) Vitamin E in the binding pocket of tubulin (PDB ID: 1SA0); Figure S8. 2D interactions of the best fit found for (A) Loliolide, (B) Ethyl linolenate, (C) Methyl linoleate, (D) Erucamide, (E) 2-Palmitoylglycerol, (F) Methyl palmitate, (G) Methyl stearate, (H) Neophytadiene and (I) Phytol docked to GlcN-6-P synthase (PDB ID: 1XFF); Figure S9. Best ranked fit of (A) Loliolide, (B) Ethyl linolenate, (C) Methyl linoleate, (D) Erucamide, (E) 2-Palmitoylglycerol, (F) Methyl palmitate, (G) Methyl stearate, (H) Neophytadiene and (I) Phytol in the binding pocket of GlcN-6-P synthase (PDB ID: 1XFF); Figure S10. (A) Best fit and (D) 2D interaction diagram of Kanamycin docked at the binding pocket of GlcN-6-P synthase (PDB ID: 1XFF). (B) Best fit and (E) 2D interaction diagram of Levamisole docked at the binding pocket of tubulin (PDB ID: 1SA0). (C) Best fit and (F) 2D interaction diagram of Loperamide docked at the binding pocket of M3 muscarinic acetylcholine receptor (PDB ID: 4U14).

## Abbreviations

EEOR	Ethanol extract of *Ophiorrhiza rugosa* leaves
p.o.	per oral
i.p.	Intraperitoneal
ANOVA	Analysis of variance
BW	body weight
SEM	standard error of mean
SPSS	statistical package for social science
ADME/T	Absorption, Distribution, Metabolism, Excretion, and Toxicity
PASS	Prediction of Activity Spectra for Substances
