# Supplementary material for: Investigation of the Biological Activities and Characterization of Bioactive Constituents of Ophiorrhiza rugosa var. prostrata (D.Don) & Mondal Leaves through In Vivo, In Vitro, and In Silico Approaches
Source: Molecules. 2019 Apr 8;24(7):1367. doi: 10.3390/molecules24071367 (PMC6480688; doi:10.3390/molecules24071367)
Supplement: Supplementary file 1 [file molecules-24-01367-s001.pdf]

Supplementary Material

Article

Investigation of the Biological Activities and Characterization of Bioactive Constituents of *Ophiorrhiza rugosa* var. *prostrata* (D.Don) & Mondal Leaves through In Vivo, In Vitro, and In Silico Approaches

Md. Adnan [1,2,†], Md. Nazim Uddin Chy [3,4,†], A.T.M. Mostafa Kamal [3,*], Md Obyedul Kalam Azad [1,5], Arkajyoti Paul [4,6], Shaikh Bokhtear Uddin [7], James W. Barlow [8], Mohammad Omar Faruque [7], Cheol Ho Park [1] and Dong Ha Cho [1,*]

[1] Department of Bio-Health Technology, Kangwon National University, Chuncheon 24341, Korea; mdadnan1991.pharma@gmail.com (M.A.); azadokalam@gmail.com (M.O.K.A.); chpark@kangwon.ac.kr (C.H.P.)

[2] Senior Scientist, Rentia Plant Factory, Chuncheon 24341, Korea

[3] Department of Pharmacy, International Islamic University Chittagong, Chittagong 4318, Bangladesh; nazim107282@gmail.com

[4] Drug Discovery, GUSTO A Research Group, Chittagong 4000, Bangladesh; arka.bgctub@gmail.com (A.P.)

[5] Head of Research and Technology, Rentia Plant Factory, Chuncheon 24341, Korea

[6] Department of Microbiology, Jagannath University, Dhaka 1100, Bangladesh

[7] Ethnobotany and Pharmacognosy Lab, Department of Botany, University of Chittagong, Chittagong 4331, Bangladesh; bokhtear@cu.ac.bd (S.B.U.); omf@cu.ac.bd (M.O.F.)

[8] Department of Chemistry, Royal College of Surgeons in Ireland, Dublin, D02YN77, Ireland; jambarlow@rcsi.ie

† These authors contributed equally to this work

* Correspondence: mostafa@pharm.iiuc.ac.bd (A.T.M.M.K.); chodh@kangwon.ac.kr (D.H.C.)

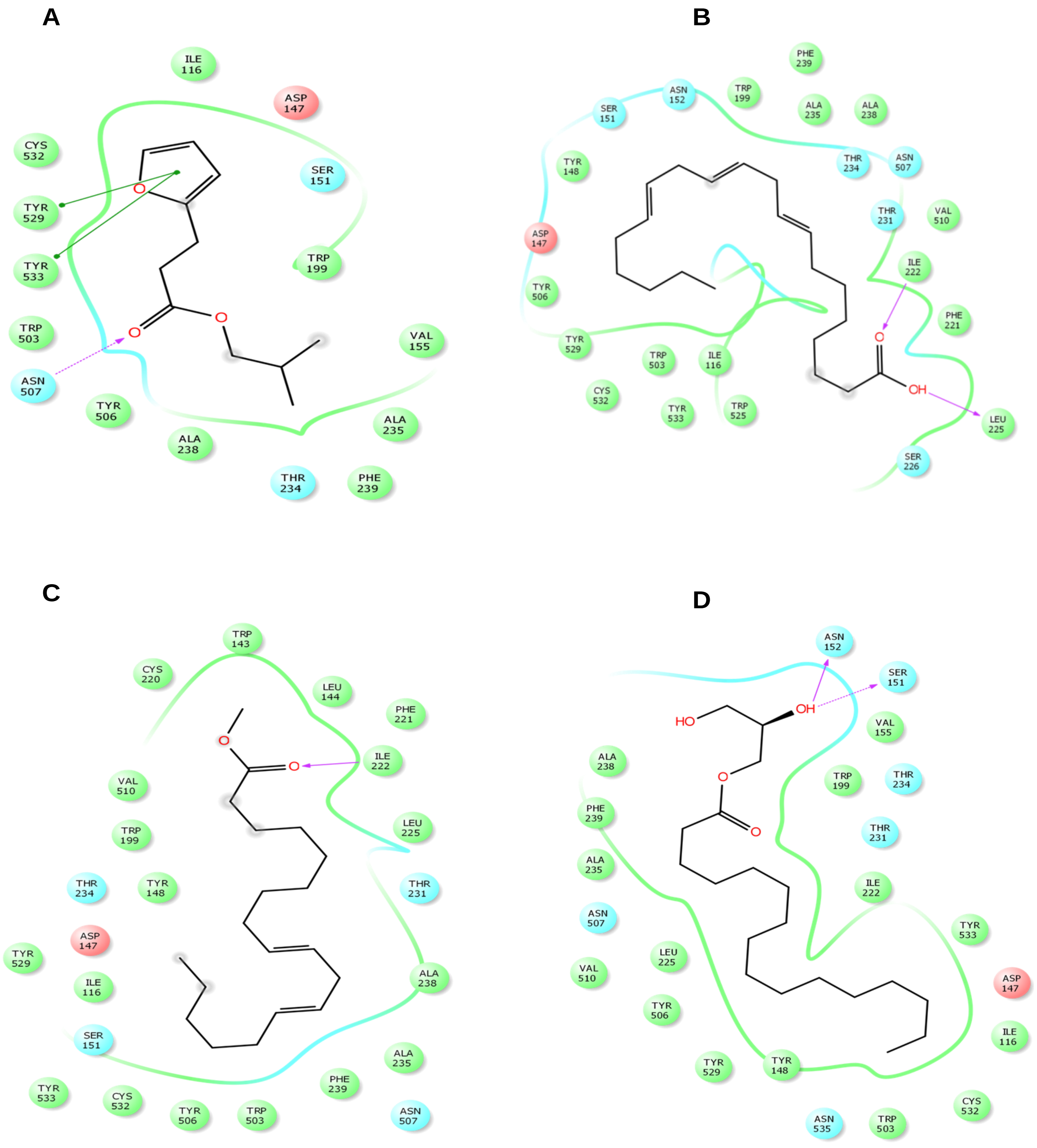
A
ILE 116
ASP 147
CYS 532
SER 151
TYR 529
TYR 533
TRP 199
TRP 503
VAL 155
ASN 507
TYR 506
ALA 235
ALA 238
THR 234
PHE 239
B
PHE 239
ASN 152
TRP 199
SER 151
ALA 235
ALA 238
TYR 148
THR 234
ASN 507
THR 231
VAL 510
ASP 147
ILE 222
TYR 506
PHE 221
TYR 529
TRP 503
ILE 116
OH
CYS 532
TYR 533
TRP 525
LEU 225
SER 226
C
TRP 143
CYS 220
LEU 144
PHE 221
ILE 222
VAL 510
LEU 225
TRP 199
THR 234
TYR 148
THR 231
ASP 147
TYR 529
ILE 116
ALA 238
SER 151
ALA 235
PHE 239
TYR 533
CYS 532
TYR 506
TRP 503
ASN 507
D
ASN 152
SER 151
HO
OH
VAL 155
ALA 238
TRP 199
THR 234
PHE 239
THR 231
ALA 235
ILE 222
ASN 507
TYR 533
LEU 225
ASP 147
VAL 510
TYR 506
ILE 116
TYR 529
TYR 148
CYS 532
ASN 535
TRP 503

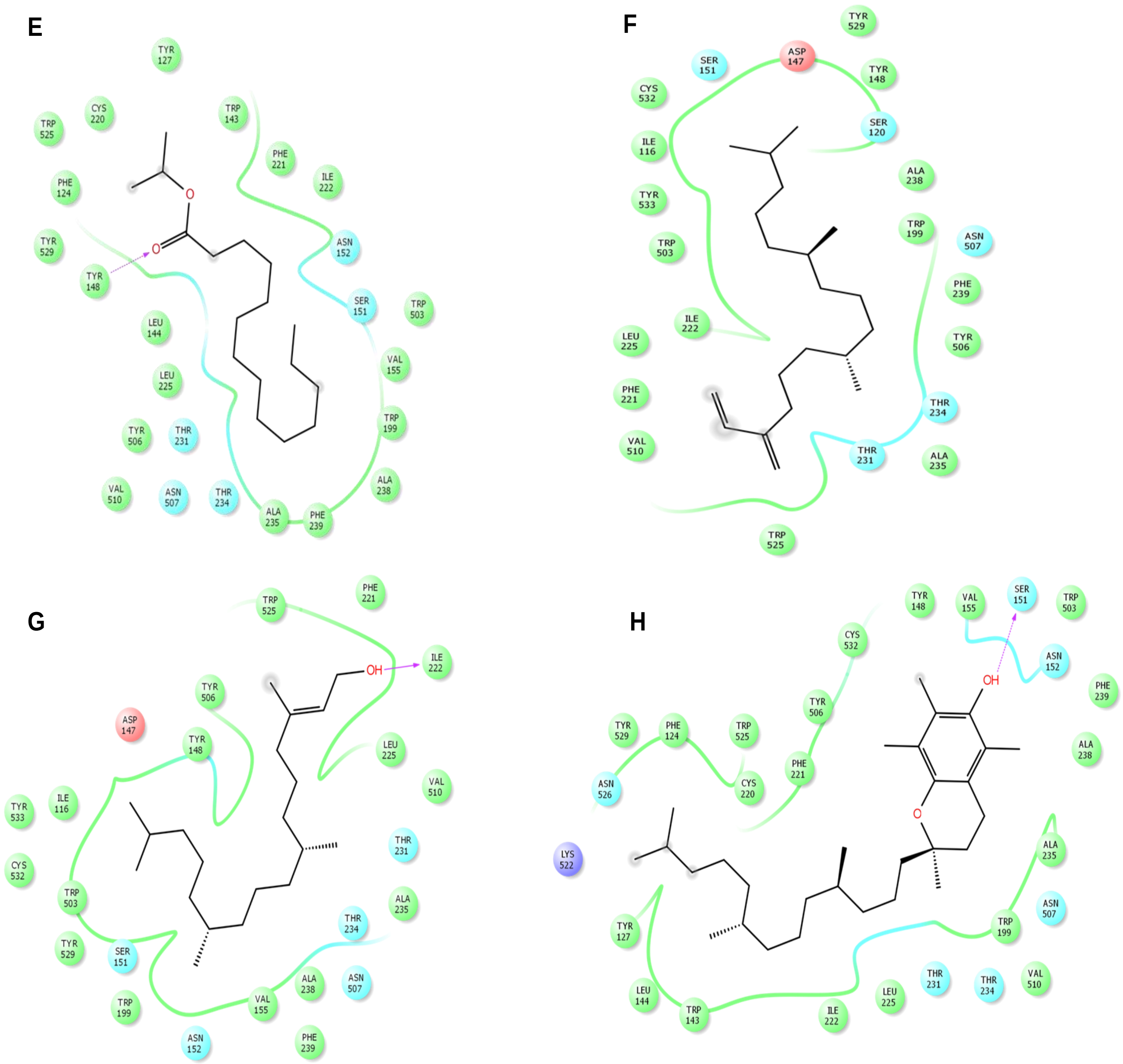

Figure S1. 2D interactions of the best fit found for (A) Loliolide, (B) Ethyl linolenate, (C) Methyl linoleate, (D) 2-Palmitoylglycerol, (E) Methyl palmitate, (F) Neophytadiene, (G) Phytol, and (H) Vitamin E docked to the M3 muscarinic acetylcholine receptor (PDB ID: 4U14). Colors indicate the residue (or species) type: Red-acidic (Asp, Glu), Green-hydrophobic (Ala, Val,Ile, Leu, Tyr, Phe, Trp, Met, Cys, Pro), Purple-basic (Hip, Lys, Arg), Blue-polar (Ser, Thr, Gln, Asn, His, Hie, Hid), Light gray-other (Gly, water) and Darker gray-metal atoms. Interactions with the protein are marked with lines between ligand atoms and protein residues: Solid pink: H-bonds to the protein backbone, Dotted pink: H-bonds to protein side chains, Green: pi-pi stacking interactions, Orange: pi-cation interactions. Ligand atoms exposed to solvent are marked with gray spheres. The protein “pocket” is displayed with a line around the ligand, colored with the color of the nearest protein residue. The gap in the line shows the opening of the pocket.

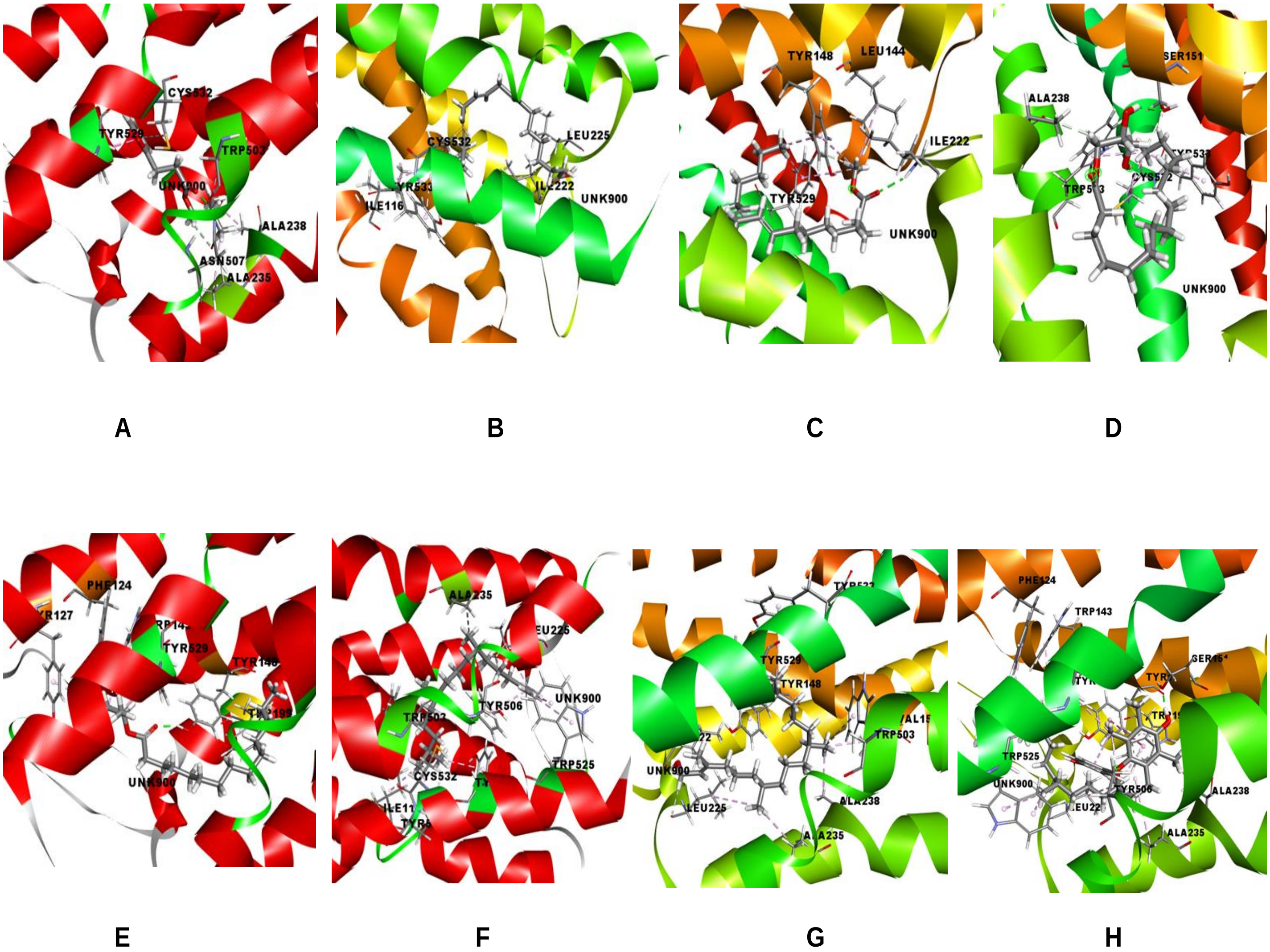

Figure S2. Best ranked fit of (A) Loliolide, (B) Ethyl linolenate, (C) Methyl linoleate, (D) 2-Palmitoylglycerol, (E) Methyl palmitate, (F) Neophytadiene, (G) Phytol and (H) Vitamin E in the binding pocket of the M3 muscarinic acetylcholine receptor (PDB ID: 4U14).

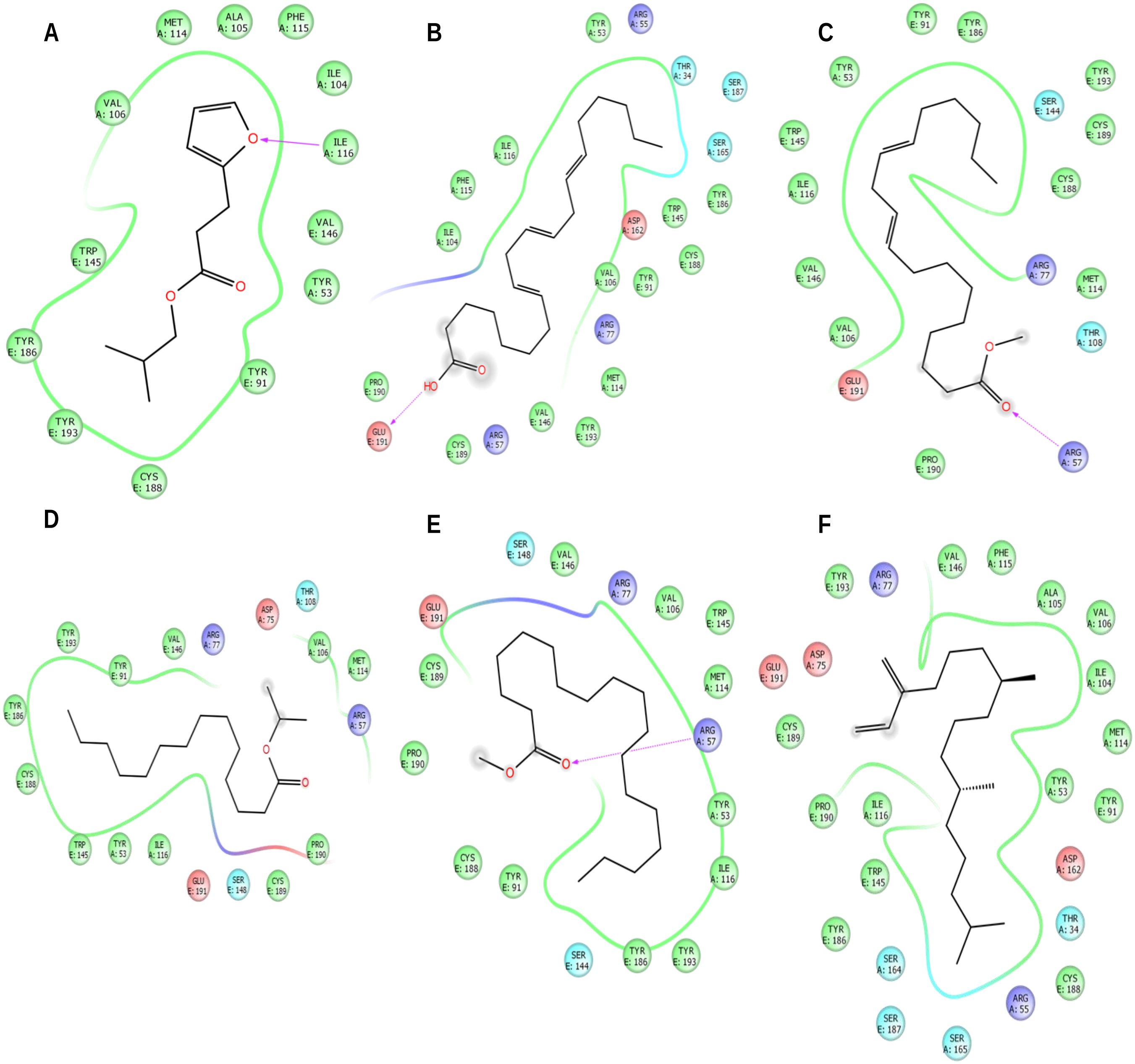

Figure S3. 2D interactions of the best fit found for (A) Loliolide, (B) Ethyl linolenate, (C) Methyl linoleate, (D) Methyl palmitate, (E) Methyl stearate and (F) Neophytadiene docked to the 5-HT3 receptor (PDB ID: 5AIN).

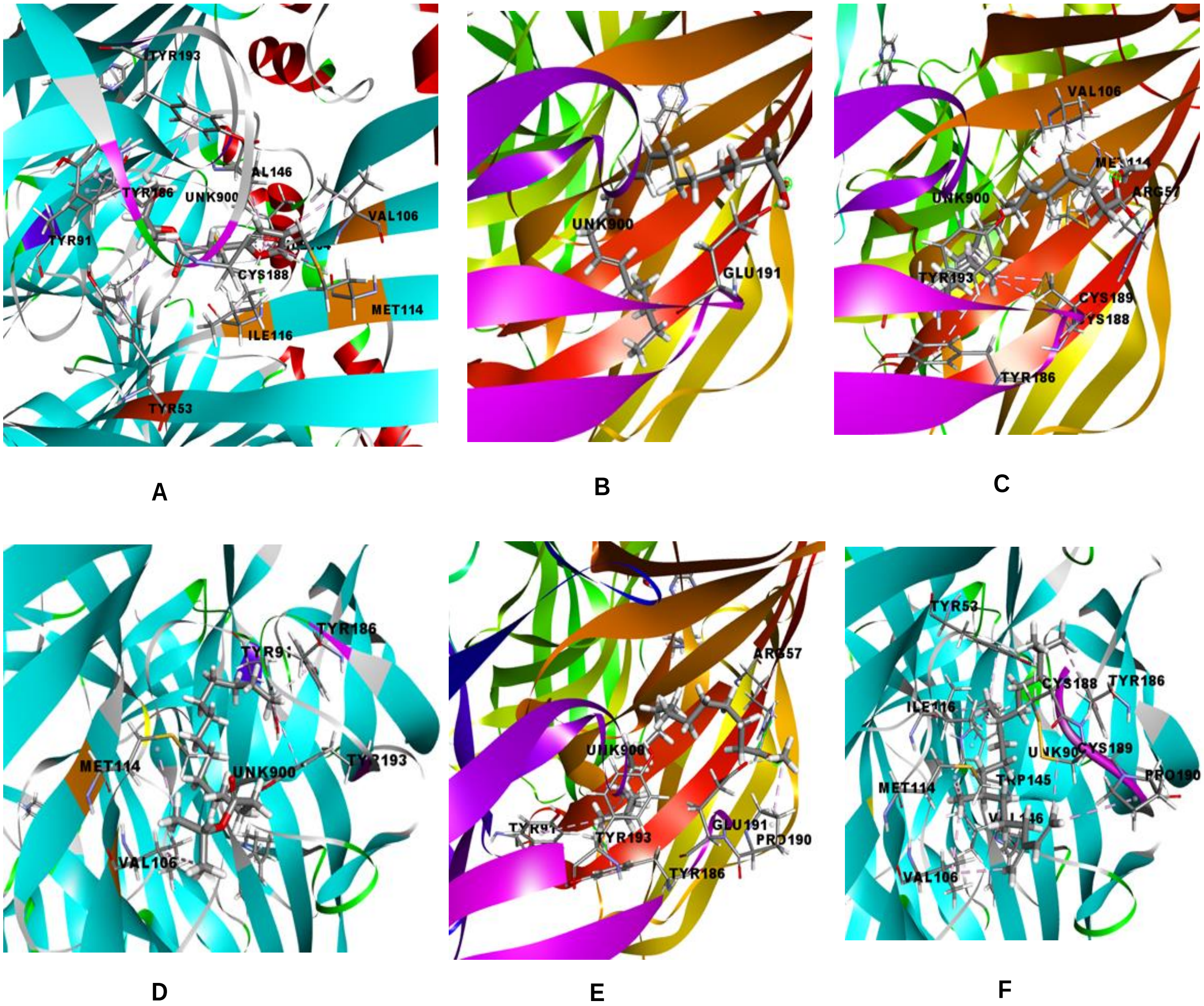

Figure S4. Best ranked fit of (A) Loliolide, (B) Ethyl linolenate, (C) Methyl linoleate, (D) Methyl palmitate, (E) Methyl stearate and (F) Neophytadiene in the binding pocket of the 5-HT3 receptor (PDB ID: 5AIN).

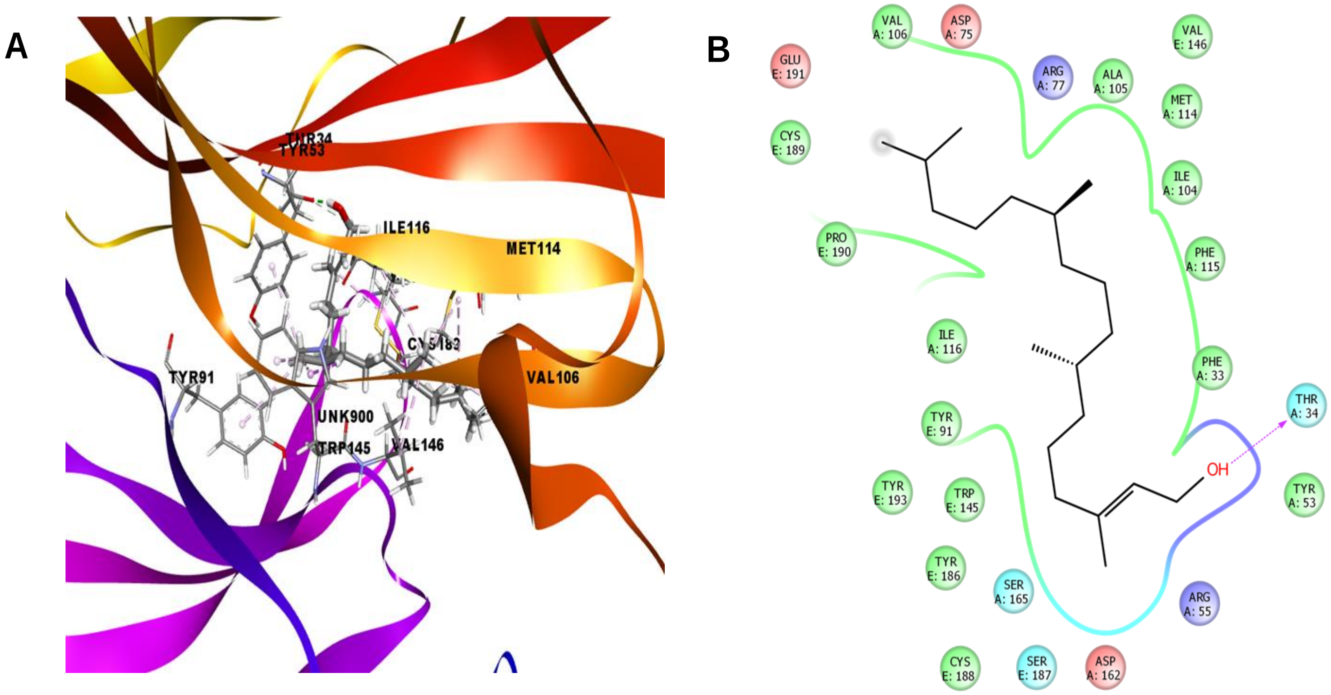

Figure S5. Best ranked fit of Phytol (A) in the binding pocket of 5-HT3 (PDB ID: 5AIN) and 2D representation of key interactions in the binding pocket for Phytol (B).

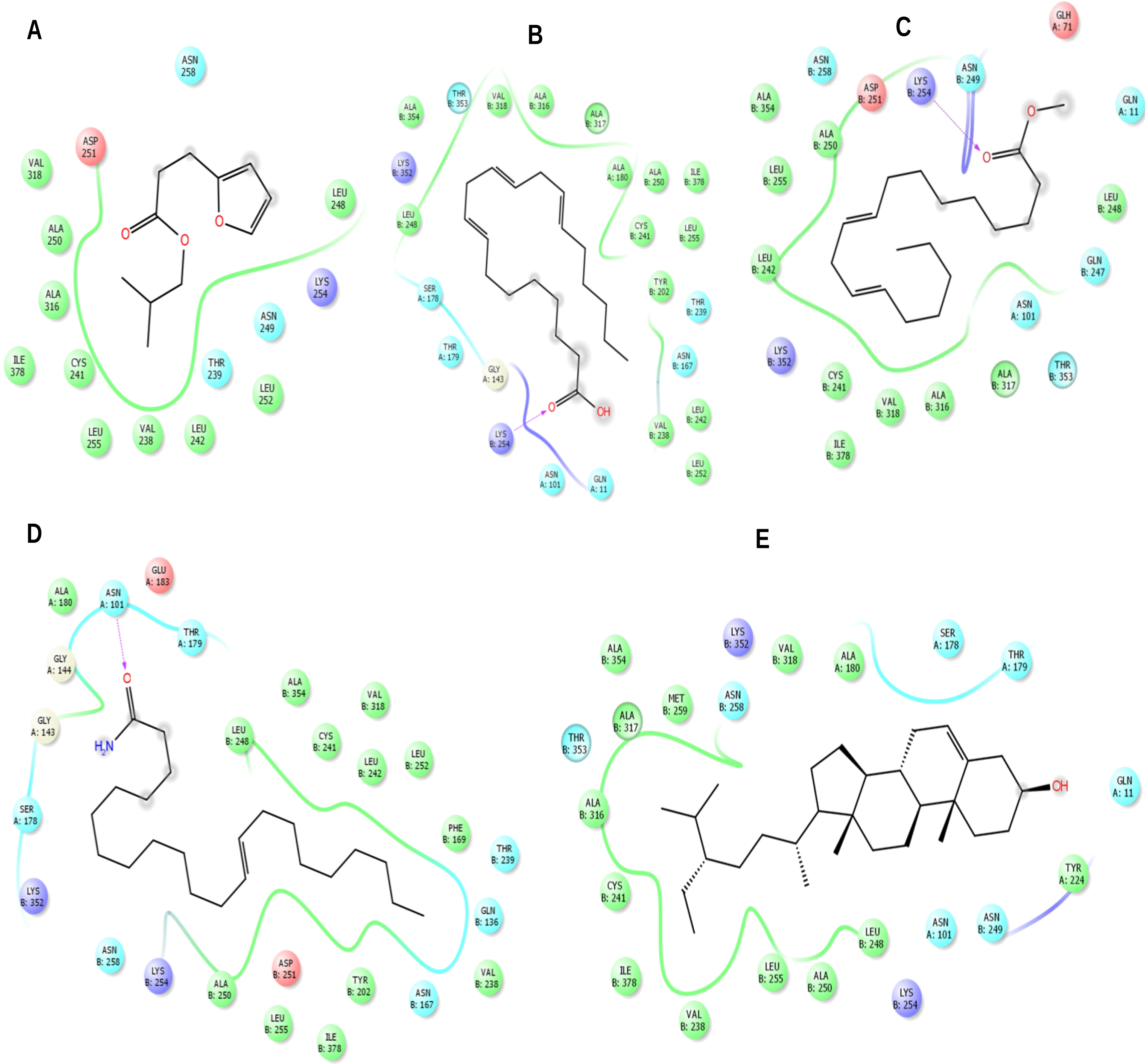
A
ASN 258
ASP 251
VAL 318
ALA 250
ALA 316
ILE 378
CYS 241
LEU 255
VAL 238
LEU 242
THR 239
LEU 252
ASN 249
LYS 254
LEU 248
O
O
O
B
ALA B: 354
THR B: 353
VAL B: 318
ALA B: 316
ALA B: 317
LYS B: 352
LEU B: 248
ALA A: 180
ALA B: 250
ILE B: 378
CYS B: 241
LEU B: 255
TYR B: 202
THR B: 239
SER A: 178
THR A: 179
GLY A: 143
LYS B: 254
ASN A: 101
GLN A: 11
ASN B: 167
LEU B: 242
VAL B: 238
LEU B: 252
O
OH
C
GLH A: 71
ASN B: 258
ASP B: 251
LYS B: 254
ASN B: 249
GLN A: 11
ALA B: 354
ALA B: 250
LEU B: 255
LEU B: 242
LEU B: 248
GLN B: 247
ASN A: 101
LYS B: 352
CYS B: 241
VAL B: 318
ALA B: 316
ALA B: 317
THR B: 353
ILE B: 378
O
O
D
ALA A: 180
ASN A: 101
GLU A: 183
THR A: 179
GLY A: 144
GLY A: 143
SER A: 178
LYS B: 352
ALA B: 354
VAL B: 318
LEU B: 248
CYS B: 241
LEU B: 242
LEU B: 252
PHE B: 169
THR B: 239
GLN B: 136
VAL B: 238
ASN B: 167
TYR B: 202
ASP B: 251
ALA B: 250
LYS B: 254
ASN B: 258
LEU B: 255
ILE B: 378
O
H2N
E
ALA B: 354
LYS B: 352
VAL B: 318
ALA A: 180
SER A: 178
THR A: 179
MET B: 259
ASN B: 258
ALA B: 317
THR B: 353
ALA B: 316
CYS B: 241
ILE B: 378
VAL B: 238
LEU B: 255
ALA B: 250
LEU B: 248
LYS B: 254
ASN A: 101
ASN B: 249
TYR A: 224
GLN A: 11
OH

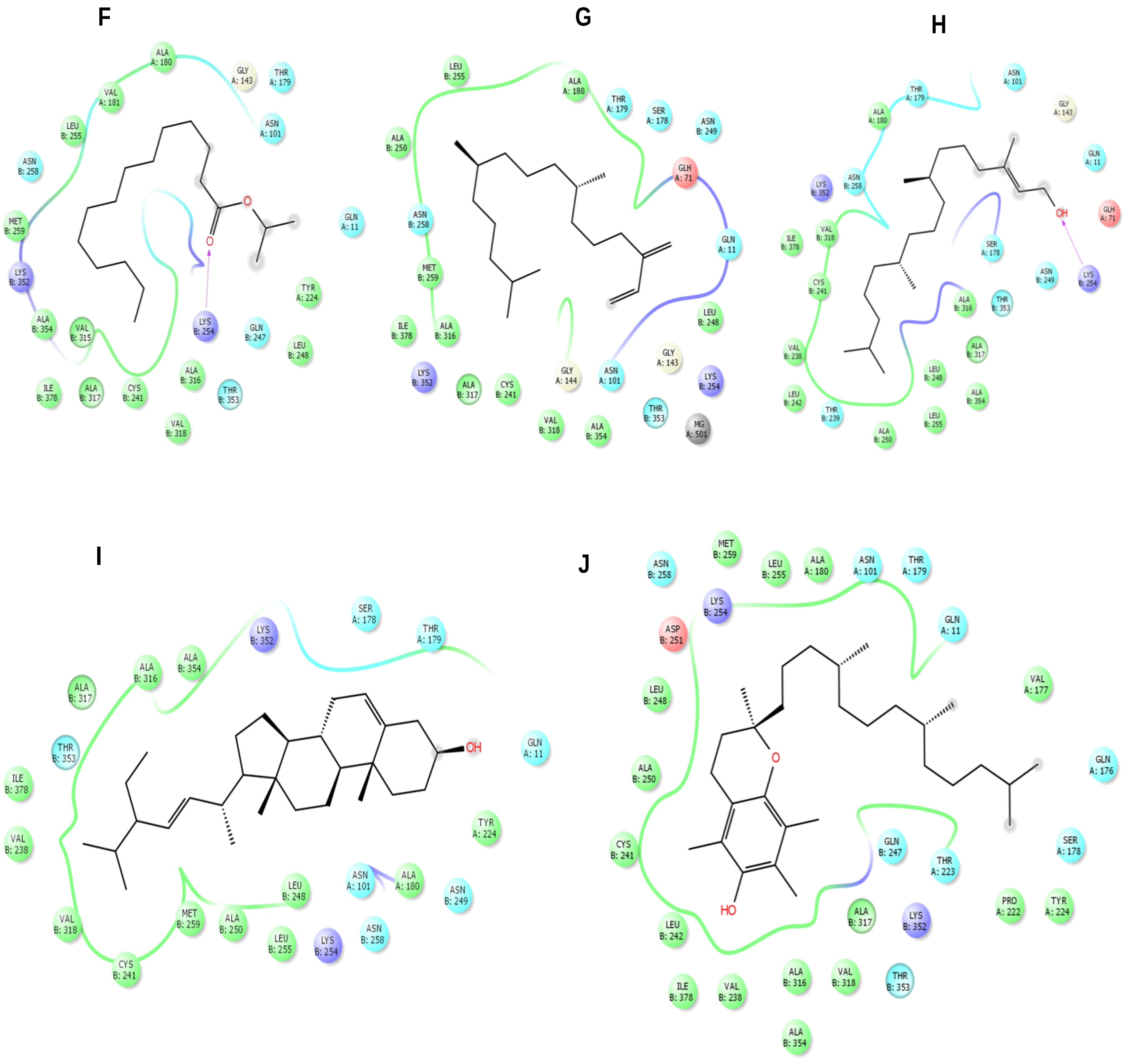

Figure S6. 2D interactions of the best fit found for (A) Loliolide, (B) Ethyl linolenate, (C) Methyl linoleate, (D) Erucamide, (E) gamma-Sitosterol, (F) Methyl palmitate, (G) Neophytadiene, (H) Phytol, (I) Stigmasterol and (J) Vitamin E docked to tubulin (PDB ID: 1SA0).

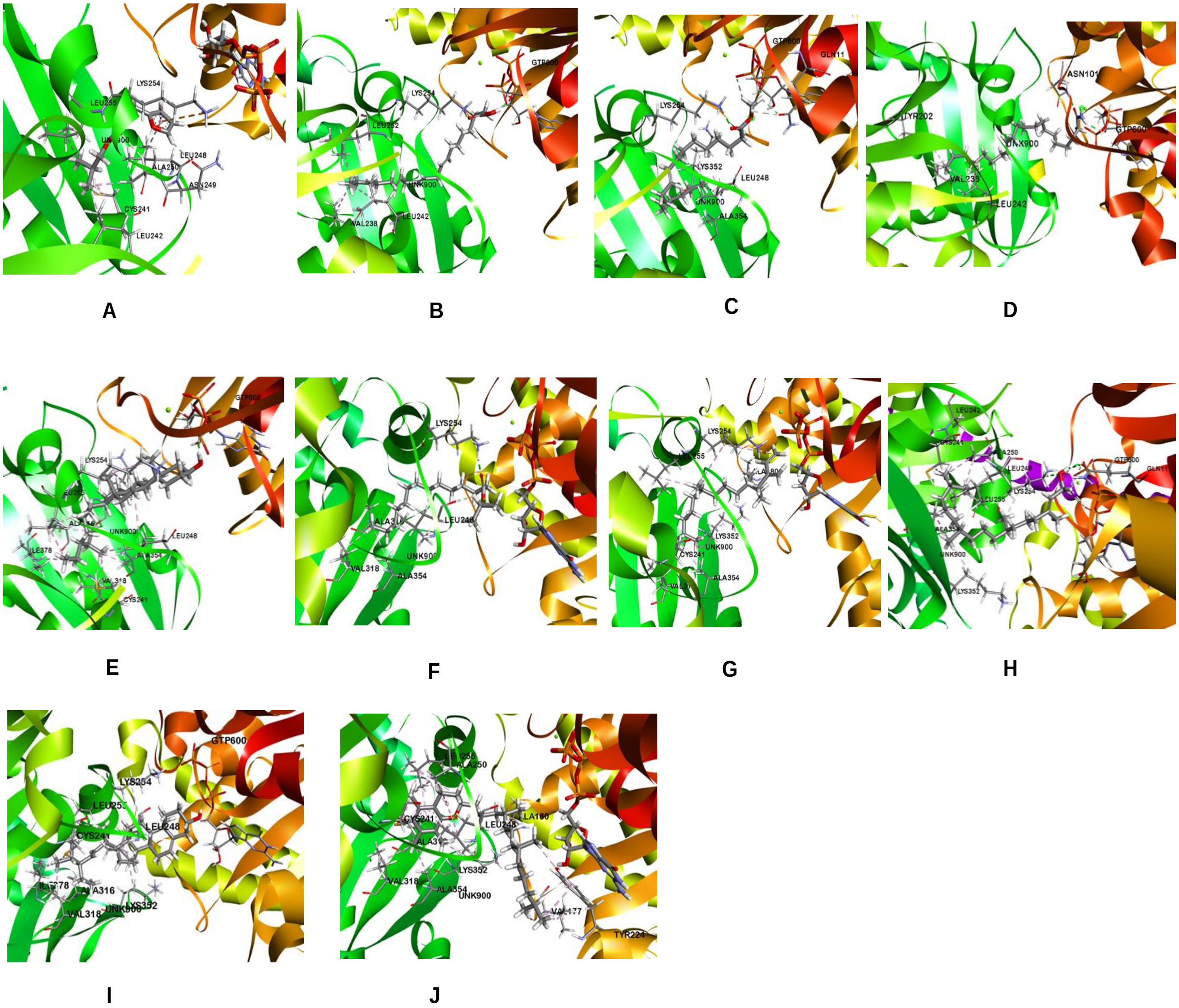

Figure S7. Best ranked fit of (A) Loliolide, (B) Ethyl linolenate, (C) Methyl linoleate, (D) Erucamide, (E) gamma-Sitosterol, (F) Methyl palmitate, (G) Neophytadiene, (H) Phytol, (I) Stigmasterol and (J) Vitamin E in the binding pocket of tubulin (PDB ID: 1SA0).

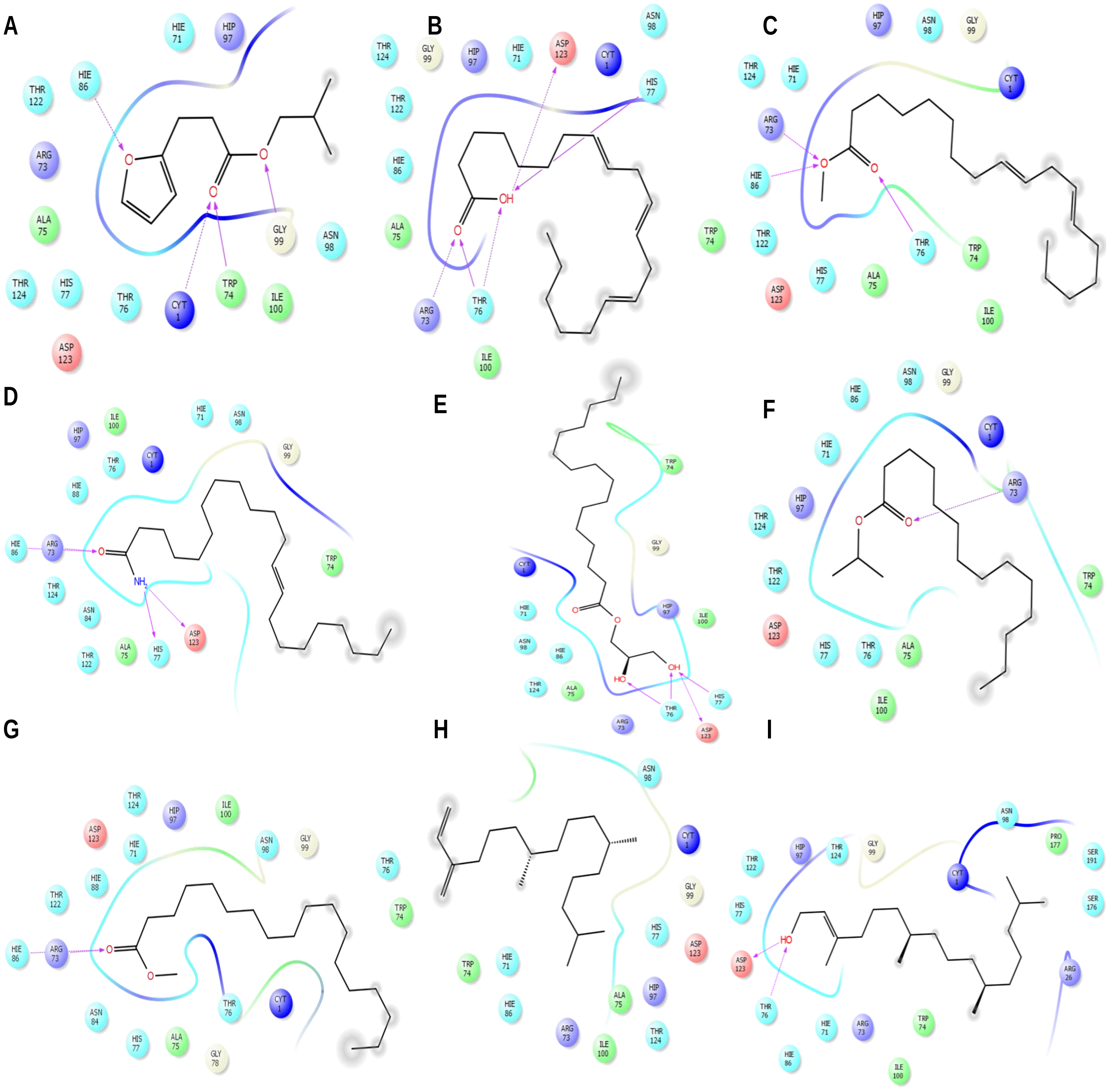

Figure S8. 2D interactions of the best fit found for (A) Loliolide, (B) Ethyl linolenate, (C) Methyl linoleate, (D) Erucamide, (E) 2-Palmitoylglycerol, (F) Methyl palmitate, (G) Methyl stearate, (H) Neophytadiene and (I) Phytol docked to GlcN-6-P synthase (PDB ID: 1XFF).

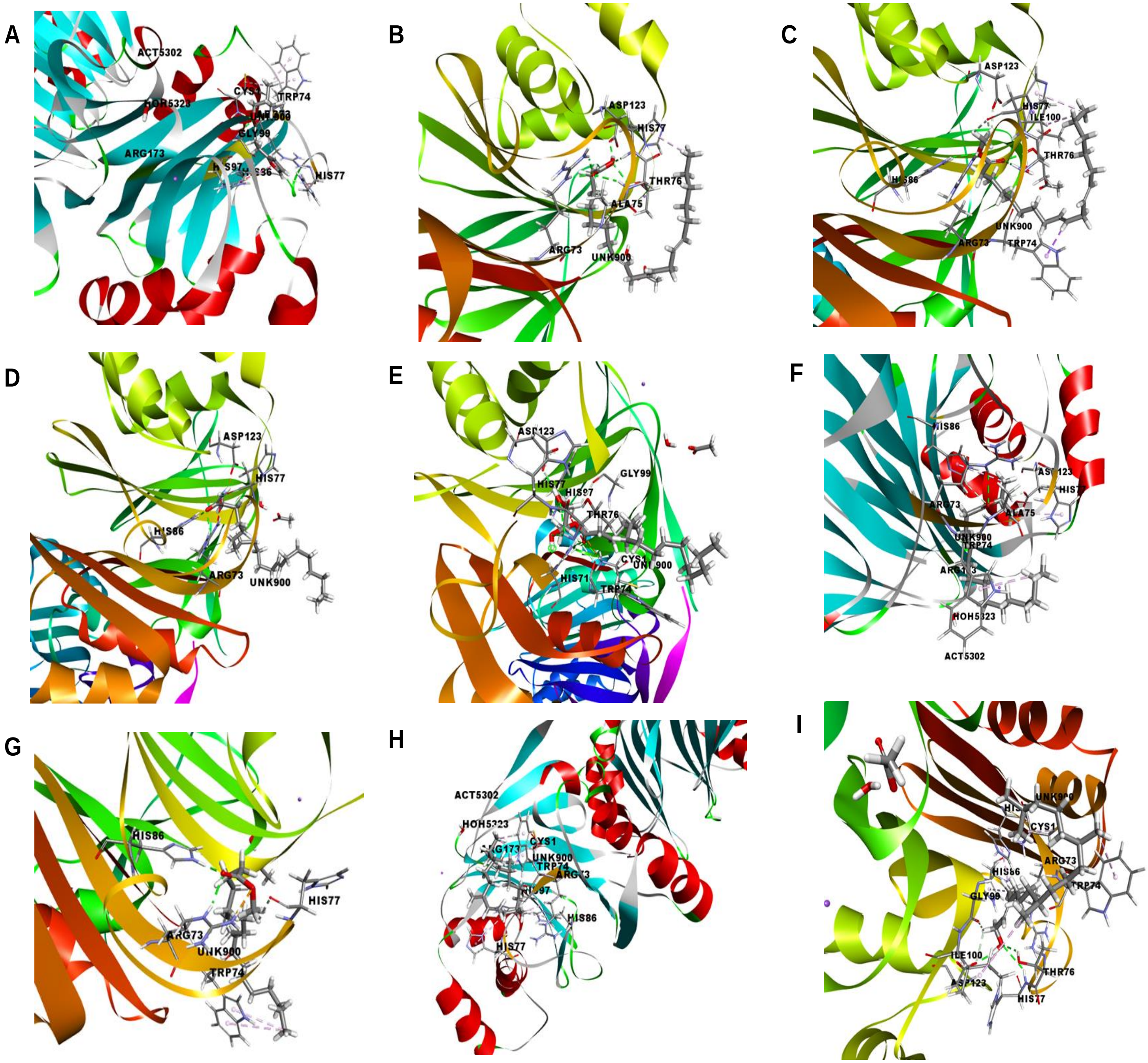

Figure S9. Best ranked fit of (A) Loliolide, (B) Ethyl linolenate, (C) Methyl linoleate, (D) Erucamide, (E) 2-Palmitoylglycerol, (F) Methyl palmitate, (G) Methyl stearate, (H) Neophytadiene and (I) Phytol in the binding pocket of GlcN-6-P synthase (PDB ID: 1XFF).

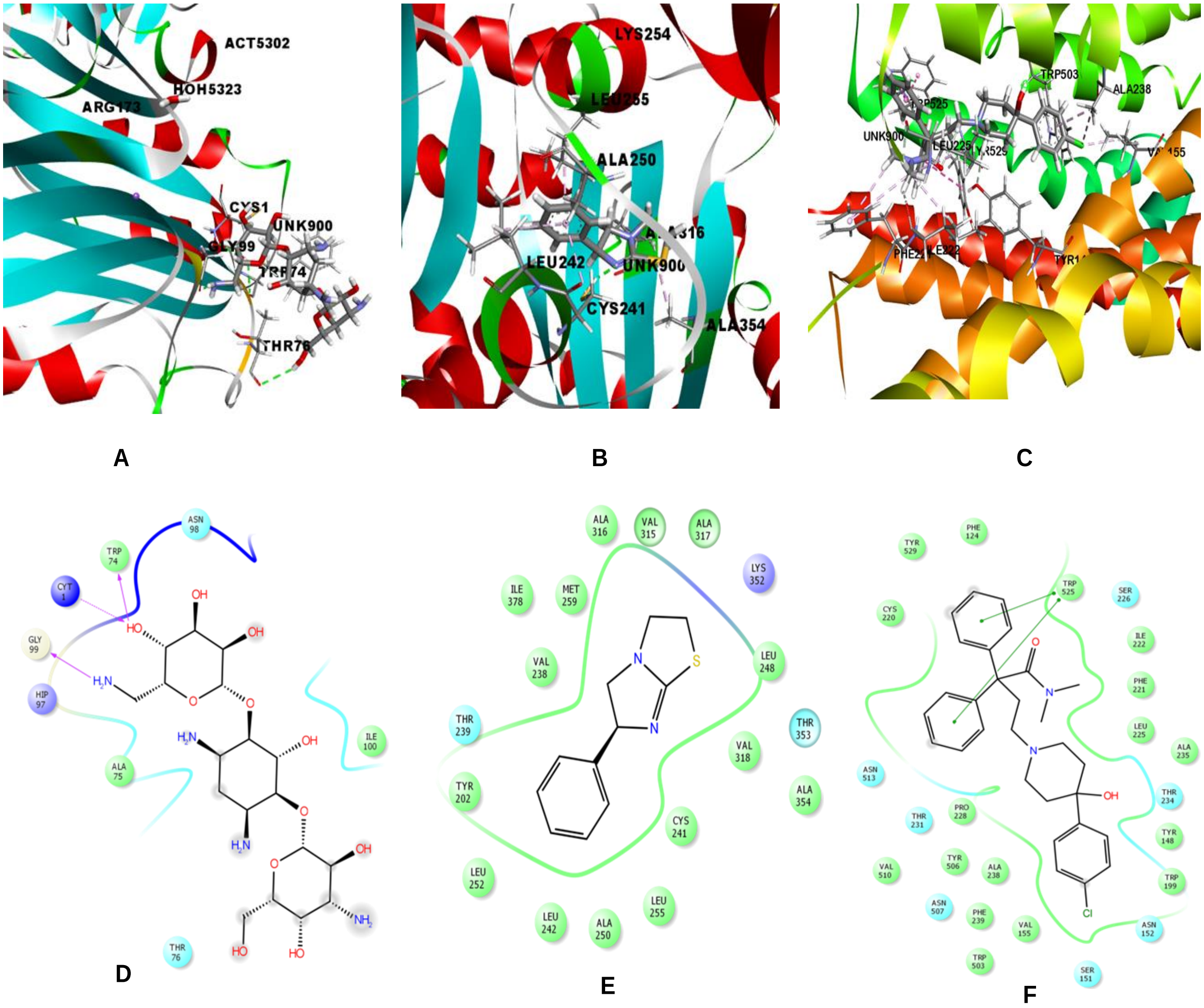

Figure S10. (A) Best fit and (D) 2D interaction diagram of Kanamycin docked at the binding pocket of GlcN-6-P synthase (PDB ID: 1XFF). (B) Best fit and (E) 2D interaction diagram of Levamisole docked at the binding pocket of tubulin (PDB ID: 1SA0). (C) Best fit and (F) 2D interaction diagram of Loperamide docked at the binding pocket of M3 muscarinic acetylcholine receptor (PDB ID: 4U14).
